# Cultural significance of medicinal plants in healing human ailments among Guji semi-pastoralist people, Suro Barguda District, Ethiopia

**DOI:** 10.1186/s13002-021-00487-4

**Published:** 2021-10-18

**Authors:** Mersha Ashagre Eshete, Ermias Lulekal Molla

**Affiliations:** 1 Department of Biology, College of Natural and Computational Sciences, Bule Hora University, P.O. Box 144, Bule Hora, Ethiopia; 2 Department of Plant Biology and Biodiversity Management, College of Natural and Computational Sciences, Addis Ababa University, P.O. Box 34731, Addis Ababa, Ethiopia

**Keywords:** Human ailments, Indigenous knowledge, Medicinal plants, Suro Barguda District

## Abstract

**Background:**

Traditional medicine has remained the most affordable and easily accessible source of treatment in the primary healthcare system among communities unable to get modern medication. Ethiopian indigenous people have a long history of traditional plant utilization for treating ailments. The objectives of this study were to identify, document, and analyze the cultural significances of medicinal plants and their associated indigenous knowledge among Guji Semi-Pastoralist People, in Suro Barguda District, West Guji Zone, southern Ethiopia.

**Methods:**

Semi-structured interview, focus group discussions, participant observation, and walk-in-the-woods methods were used to gather medicinal plants data. The informant consensus factor (ICF) and fidelity level (FL) values were calculated using quantitative approaches to check the level of informants' agreement on plant use and the healing potential of medicinal plant species, respectively. Indigenous knowledge of the use of medicinal plants for medicinal purposes among different informant groups was compared using *t* tests with R software.

**Results:**

A total of 98 medicinal plant species belonging to 87 genera and 48 families were reported to be used for treating human ailments such as gastrointestinal diseases, breathing system diseases, dermatological diseases, and febrile diseases. Family Fabaceae was represented by 10 species followed by Lamiaceae (7 species). Four of the medicinal plants (*Bothriocline schimperi* Oliver & Hiern ex Bentham, *Erythrina brucei* Schweinf. emend. Gillett, *Lippia adoensis* Hochst. ex Walp. var. *adoensis,* and *Millettia ferruginea* (Hochst.) Hochst. ex Baker) were found endemic to Ethiopia and shrubs were more dominant (36 species). Ninety-one medicinal plant species were used for remedy preparation as soon as they were collected in their fresh form; 35.6% herbal medicine preparation was through crushing the plant parts and homogenizing them with cold and clean water; 159 (70.4%) traditional medicinal preparations were reported to be taken in their drinking form (orally).

**Conclusion:**

The study indicated that the district is rich in different species of medicinal plants used to treat human ailments and indigenous knowledge about using these resources. Species with the recorded highest consensus for curative purposes are useful sources for further phytochemical and pharmacological validation for better utilization. Declining wild medicinal flora of the area calls for conservation priority.

**Supplementary Information:**

The online version contains supplementary material available at 10.1186/s13002-021-00487-4.

## Background

Ethnobotany is an academic discipline defined as “local people's interaction with the natural environment: how they classify, manage and use plants available around them” [1]. Medicinal plants have been used in health care since time immemorial. Studies have been carried out globally to verify their efficacy, and some of the findings have led to the production of plant-based medicines. Traditional medicine includes the diversity of health practices, approaches, knowledge, and beliefs incorporating plant, animal, and/or mineral-based medicines, spiritual therapies, manual techniques, and exercises, applied singly or in combination to maintain well-being through treating, diagnosing, or preventing illnesses [2]. The inclusiveness of the term “traditional medicine” and the wide range of practices it encompasses make it difficult to define or describe, especially in a global context. Traditional medical knowledge may be passed on orally from generation to generation, in some cases with families specializing in specific treatments. Sometimes, its practice is quite restricted geographically, and it may also be found in diverse regions of the world. However, in most cases, a medical system is called “traditional” when it is practiced within the country of origin. Indigenous/traditional knowledge has developed as a result of human interaction with their environment. Traditional knowledge related to the health of humans and animals exists in all African countries. Every region has had, at one time in its history, a form of traditional medicine. Each African community has its own particular approach to health and disease, even at the level of ethnopathogenic perceptions of diseases and therapeutic behavior. Traditional healers and remedies made from plants play an important role in the health of millions of people. So, ethnobotanical studies are useful in documenting, analyzing, and communicating knowledge and interaction between plant diversity and human societies, how diversity in nature is used and influenced by human activities [1–3]. In discussing the many potential uses and ways of interacting with local plants, we anticipated expressing a sense of the value of the study area landscapes with the associated vegetation and respective indigenous knowledge. Traditional knowledge is coming into the middle-of-the-road for sustainable development and biodiversity conservation discussion. Indigenous knowledge guides the choices and practices of pastoralists and farmers of many places, and predictably, some 80% of the world's population fulfills their primary health needs through the use of traditional medicines [4]. Even in developed countries, local knowledge built up across generations continues to play a fundamental role in supporting localized resource use practices whether they are pastoralists, small-scale farmers, or the gatherers of wild produce. Indigenous people can contribute importantly to the understanding of the processes of change, whether these might be short or long term which can be bounded to local events or global transformation processes. Pastoral and peasant communities that have maintained traditional modes of production have today become the major guardians of the world's crop and domestic animal diversity. However, overstocking and farmland expansion has become the main causes of natural resource degradation. Despite these, studies on the ethnobotany of the woodland and dry Afromontane vegetation in Suro Barguda District are lacking. So, it was important to study the diversity of medicinal plants and associated indigenous knowledge in the study area to determine the level of their usage, depletion/conservation. So, the objectives of this study were to identify, document, and analyze the significance of medicinal plants and their associated indigenous knowledge in the preparation and application of the remedies by local people in the study area.

## Traditional medicinal plants

In Ethiopia, there is limited development of therapeutic products from traditional medicinal plants and the indigenous knowledge on the practice of medicinal plants is being lost owing to migration from rural to urban areas, industrialization, fast loss of natural habitats, and transformations in lifestyle. There is also a lack of adequate ethnobotanical surveys in many parts of the country. Because of these, records of the traditional use of medicinal plants are an urgent matter and important to preserve the knowledge [5, 6]. The sociocultural appeal, the cultural acceptability of healers and local pharmacopeias, accessibility, being fair in its price, and effectiveness against several health problems seem to foster its widespread use [7, 8]. The Ethiopian traditional medical system is characterized by variation and is shaped by the environmental diversities of the country, sociocultural conditions of the different ethnic groups as well as historical developments that are related to migration, the introduction of foreign culture, and religion [9–12]. Traditional medical practitioners treat people and most of the health services rendered by these practitioners are focused on communicable diseases among people. Proper management of traditional medicinal plant resources is essential, not only because of their value as a potential source of new drugs but due to reliance on traditional medicinal plants for health. Ethnobotanical studies can indicate management problems of medicinal plants through interviews and market surveys and it gives solutions by promoting local traditions and customs that had conservation merits [13, 14]. So that an inclusive compilation of medicinal plants that can be used in disease prevention is obtained, the collection of original data from the traditional guardians of such knowledge is essential [15]. This is especially so in the case of African Traditional Medicine where information is passed on from generation to generation orally about the plants used. Unlike in Chinese Traditional Medicine and the Indian systems of medicine (Ayurveda, Unani, and Sithda) where the information is available in books (and now online), a lot of the information on African traditional medicine is yet to be documented. Efforts are, however, being made by WHO, to supplement the various isolated databases on medicinal plants through the provision of guidelines for documentation of herbal recipes [16]. Considerable knowledge accumulated by the villagers and tribes on herbal medicine remains unknown to the scientist and urban people. Many plant species associated with the rural people are on the verge of disappearing and are on the vulnerable list. The impact of deforestation, urbanization, and modernization is shifting the rural people from their natural habitats and their very knowledge particularly concerning herbal drugs is slowly disappearing. Our direct concern is to preserve this knowledge. Whatever knowledge exists today is mostly confined to the older generation. In this context, some approaches needed for the preservation and development of traditional knowledge are presented here, based on the authors’ experience in an ethnomedico-botanical survey.

Therefore, people living in Suro Barguda District have traditional practices which they put into effect for generations to take care of themselves. On the other hand, the area has been losing its indigenous flora due to human and other biotic and natural causes. This loss of indigenous flora links with the missing of important indigenous knowledge connected with the plants. Hence, there is a clear need to conduct an ethnobotanical study on the diverse medicinal plants in the area, to look into and compile relevant information, and to document them before it becomes difficult to gain the knowledge of the indigenous people.

## Materials and methods

### Description of the study area

**Fig. 1:**
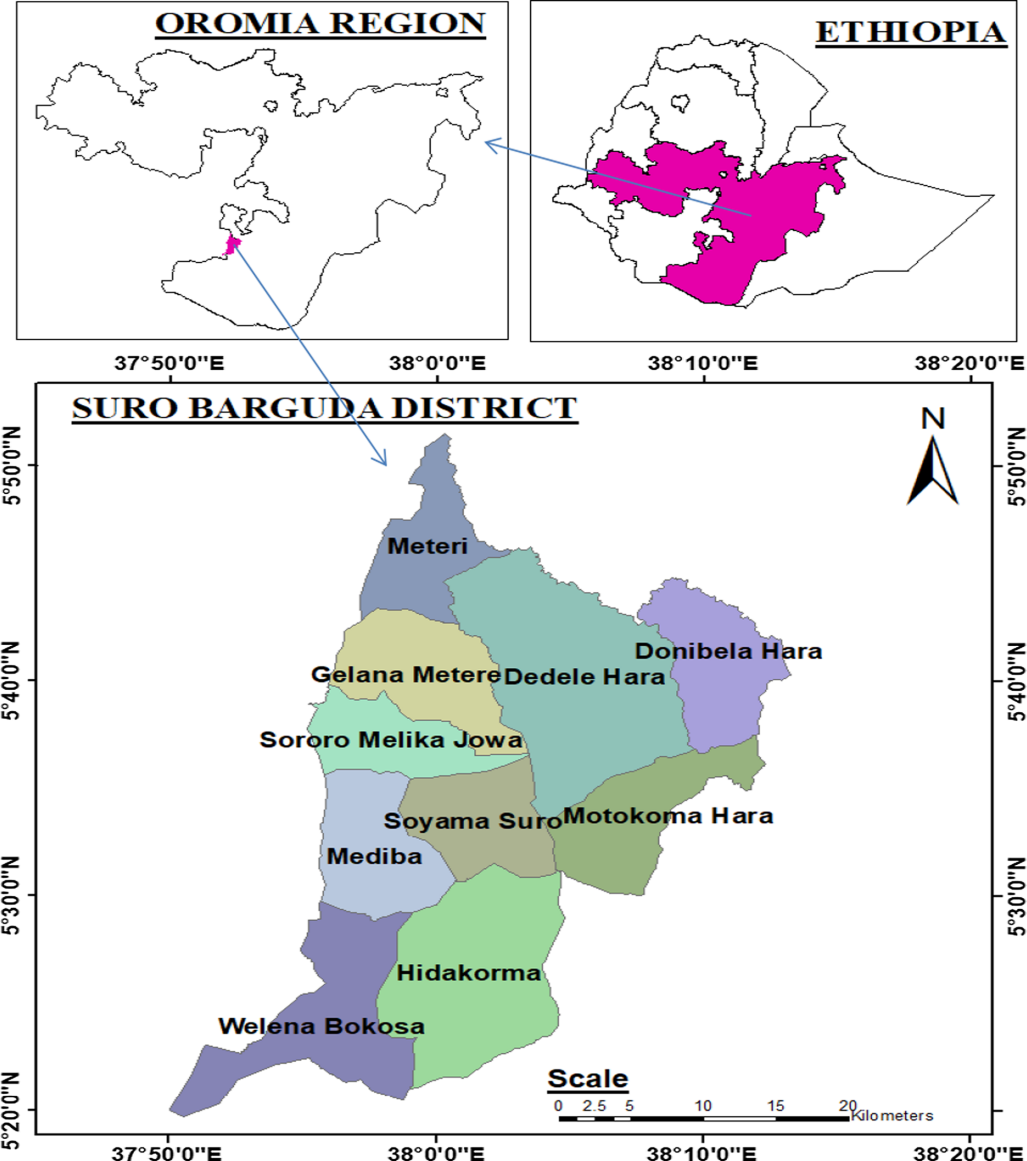
Map of Ethiopia showing Suro Barguda District (the study area)

The present study was conducted in Suro Barguda District, West Guji Zone of Oromia Regional State, southern Ethiopia. Suro Barguda District is located 497 km south of Addis Ababa, the capital of Ethiopia, and 30 km from Bule Hora town, the capital of West Guji Zone. The district is generally characterized by rough and rugged topography and lies between latitudes 5°30′0″N and 5°50′0″N, and longitudes 37°50′0″E and 38°20′0″E. The altitude ranges from 900 to 2350 m.a.s.l., and the total area of the district is 154,958.4 ha [17] (Fig. 1).

Suro Barguda District is divided into two agroecological zones, namely the lowlands (from 900 to 1500 m a.s.l) and the middle altitude ranging from 1501 to 2500 m a.s.l. [18]. Accordingly, the proportion of the two agro-climatic zones in the district is 41.8% lowlands and 58.2% mid-altitude. This district falls within the southern bimodal rainfall regime of Ethiopia. Since there was no Meteorological Station at Suro Barguda District, 15 years of Meteorological data (2004–2018) registered by the nearby Station (Bule Hora District Station) was taken from National Meteorological Service Agency. Based on the analysis of these data, the district receives high rainfall between March and half of June as well as a relatively good amount from half of August to half of November. The dry season extends from December to February and some extent from half of June to half of August. The highest mean annual average rainfall of the study area within 15 years was 171.3 mm recorded in May, whereas the lowest mean average was 12.4 mm recorded in February. The lowest mean average temperature over 15 years was 10.8 °C recorded in December, whereas the highest was 28.7 °C recorded in February. The mean annual rainfall of the study area was 853 mm, whereas the mean annual temperature was 19.8 °C [19]. Based on [20] classification of Ethiopian vegetation, the study area vegetation lies in the Acacia-Commiphora woodland and Bushland, Combretum-Terminalia Woodland, and Dry Evergreen Afro-montane Forest and Grassland complex.

### Demographics and human health condition in the district

Since Suro Barguda District was established recently (administrative boundaries were redrawn), a population census has not yet been formally carried out. The district is predominantly (99.9%) occupied by Guji Oromo ethnic groups who speak the Oromo language with unique dialect and the majority of the residents live in rural areas and follow a traditional belief called “Waaqqeefatta.” But nowadays some of them tend to attend protestant teaching [21]. Pastoralism with subsistence farming is the most common economic mainstay of the people. As in most rural districts of Ethiopia, access to modern health services is poor. Based on the 2016–2018 District's Health Office report, 41,030 people were assisted by modern health service which covers only 55% of the population [22]. There are two governmental health centers and eight health posts in Suro Barguda District. Concerning human health professionals in the district, there were three health officers, two general nurses, 40 clinical nurses, two laboratory technicians, one sanitarian, eight midwives, one pharmacist, 54 health extension workers, and 10 support staff. The five common health problems in the district were acute respiratory tract infection, pneumonia, typhoid, internal parasitoid, different types of cancer symptoms, and malaria [22].

### Study site selection

A reconnaissance survey of the study area was conducted from May 06–21, 2019, to obtain information about the agroecology of the area, status of the vegetation, and indigenous knowledge of the local people in using plants for different purposes and determine the sites from where and how the data should be collected. The study district had 10 semi-pastoralist kebeles (the smallest administrative units), and currently, these kebeles are rearranged/subdivided to be 19 kebeles to decentralize the administration processes. Study sites from the ten kebeles were selected based on distance from the administrative town (Suro town) and the presence/absence of health facilities for collecting medicinal plant information.

### Informant size determination

The informant size for collecting quantitative and qualitative data for medicinal plants research to ensure the required representative size of households from all semi-pastoralist kebeles followed Cochran’s (1977) formula as indicated by [23].

$$n = \frac{N}{{1 + N(e)^{2} }}$$

where *n* = sample size for the research; *N* = total number of households in all the 10 kebeles; *e* = maximum variability or margin of error 5% (0.05); and 1 = the probability of the event occurring.

The total number of households in the 10 pastoralists’ kebeles of the district was 386. Hence, the informant sample size becomes:

$$\begin{array}{*{20}l} = \hfill & {\underline{382} } \hfill & = \hfill & {\underline{382} } \hfill & { = {\text{196 informants}}} \hfill \\ {} \hfill & {1 + 386\left( {0.05} \right)^{2} } \hfill & {} \hfill & {1.965} \hfill & {} \hfill \\ \end{array}$$

Therefore, the required informant (respondent) size was 196. Informants’ size for each kebele was calculated using the amount of the number of households in each kebele to the total number of households of the 10 kebeles, i.e.,

$${\text{Informants from each kebele}} = {\text{Number of households of the kebele}} \times {\text{Total number of informants}}/{\text{Total number of households}}.$$

For example, the informant size of Dembela Hara kebele with a total household of 47 was 24, i.e., (47 × 196/386 = 24). The same calculation was used for the other study kebeles, and two to four key informants were taken purposefully from each kebele based on the size of the households (a total of 24 key informants). The 24 key informants were those informants having good recognition in treating different diseases (healers/practitioners) and selected purposively (non-probability sampling) through consulting local leaders, elders, and development agents at each kebele. The remaining 172 participants were general informants and were taken randomly to get hold of people who had no official recognition for their traditional healing practices. The general informants were ordinary people who lived in the study area for a long period of time and used their indigenous medicinal plant knowledge within their families. They were included as respondents to gather additional data and check the transfer of indigenous knowledge within the people. The participants were involved in data collection activities at two different times: from July 01 to August 30, 2019 (for 2 months), and November 15 to December 30, 2019 (for 45 days). All informants were from Guji Oromo ethnic groups who speak the Oromo language called “Oromiiffaa” (Table 1).

**Table 1:** Number of households and informants included for the ethnobotanical data collection

Name of the kebele	Total no. of hh	Key informants					Randomly taken informants					Total informants		
		M	Ag	W	Ag	T	M	Ag	W	Ag	T	M	W	T
Dembela Hara 1840–1980 m. a. s. l	47	2	62, 73	1	65	3	17	19–51	4	22–39	21	19	5	24
Didole Hara 1790–1965 m. a. s. l	43	2	59, 64	1	52	3	15	23–42	4	25–56	19	17	5	22
Gelana Meteri 1050–1825 m. a. s. l	36	2	80, 54	0	–	2	14	20–44	2	25, 50	16	16	2	18
Hidha Korma 1990–2350 m. a. s. l	37	2	78, 66	0	–	2	15	24–57	2	21, 71	17	17	2	19
Mediba 1963–2185 m. a. s. l	40	2	48, 56	1	63	3	14	22–65	3	27–47	17	16	4	20
Meteri 900–1835 m. a. s. l	31	2	52, 76	0	–	2	13	25–70	1	29	14	15	1	16
Motokoma Hara, 1973 -2050 m. a. s. l	39	2	61, 73	0	–	2	16	21–43	2	34, 38	18	18	2	20
Sororo Melka Jewe 945–1874 m. a. s. l	34	2	50, 77	0	–	2	13	26–40	2	24, 49	15	15	2	17
Soyama Suro 1982–2100 m. a. s. l	40	2	68, 75	1	58	3	15	19–41	2	26, 67	17	17	3	20
Welena Bokosa 2100–2280 m. a. s. l	39	2	65, 67	0	–	2	16	27–69	2	35, 76	18	18	2	20
Total	**386**	20	–	4	–	**24**	148	–	24	–	**172**	168	28	**196**

Bold values indicate the total of the respected column

*hh* household, *M* men, *W* women, *Ag* age, *T* total, *m. a. s. l.* meter above sea level

### Data collection

As it is mentioned above, data were collected two times: from July 01 to August 30, 2019, and November 15 to December 30, 2019, and plants reported as medicine by the informants were collected. During data collection, the researchers used motor bicycle for accessing study kebeles and informants’ activity. Ethnobotanical data were collected as follows [1, 3, 24]. Semi-structured interviews, guided field walks, discussions, market surveys, and field observation, with randomly picked and key informants, were applied based on a checklist of questions. The selected informants in the sample site were interviewed using semi-structured interview which was translated into the local language focusing on medicinal plants: their uses and management; from where they collect them; how could they manage if these plants cause negative side effect on users; which plant was more preferable in its use; how they know their habitat and time of availability; whether they got any economic benefit from medicinal plants or not; whether they had any tendency to cultivate some selected medicinal plants or not; about the level of any threat to the medicinal plants; what they suggest about the current conservation status of these plants; how widespread the medicinal plant/s in the area are; whether there was disappeared medicinal plant or not; whether there was any restriction or taboo in collecting medicinal plants or not; whether these plants had other purposes or not; etc.

A semi-structured interview questionnaire was an important tool for the collection of both qualitative and quantitative data at the same time. The informants participated in answering the questions by showing the plants that they used as medicine during the guided field walk interview. An explanatory individual and group discussion was made with the informants at each locality and site focusing on the status of the vegetation and acceptance of medicinal plants by the community. Detailed notes on facts and information about the respondents, history of medicinal plant users, status of medicinal plants, and other essential information (based on the questionnaire) were taken on site. During the discussion, the informants were free to explain medicinal plants and their knowledge without being interfered with and restricted. The collected medicinal plant species were brought to Bule Hora University Herbarium where they were allowed to dry and deep-frozen and identifications were made by the researchers using taxonomic explanations and descriptions given in the relevant volumes of the Flora of Ethiopia and Eritrea. Further refining of determinations was made by visual comparison with authenticated herbarium specimens. The plant specimens with labels were finally deposited at the mentioned Herbarium, and the resulting data of the study were presented in tables, graphs, and percentages.

### Ethnobotanical data analysis

Ethnobotanical data were analyzed following the basic analytical tools [1, 25, 26]. Potentially effective medicinal plants were identified by the method of informant consensus factor [27]. Rank ordering (preference ranking) of medicinal plants was used to determine their order of cultural importance across a community. The most important in the set was given the highest number, decreasing in number as the members of the set decrease in importance. Preference ranking was computed by taking 10 key informants to assess the degree of effectiveness of those medicinal plants highly cited by the informants used to treat a particular disease [1]. Direct matrix ranking was a more multifaceted version of preference ranking. Here informants order medicinal plants by considering several attributes one at a time, i.e., it draws explicitly upon multiple dimensions. Direct matrix ranking was performed as a group exercise in which participants reach a consensus on the ranking of each item based on their evaluations [1]. The ranking of threats on 10 medicinal plants that were reported by most of the informants in the study area was conducted using ten key informants as described by Martin [1] and Alexiades [28]. This information was used to determine the highest threats to traditional medicinal plants in the study area and help to suggest appropriate conservation measures as considered. Informant consensus factor (ICF) was considered for each group of ailments to identify the agreement of the informants on the reported cures for the group of aliments of the plant. Informant consensus factor was computed as follows: the number of use citations in each group (*n*_*ur*_) minus the number of species used (*n*_*t*_), divided by the number of use citations in each group minus one [29]. The mentioned ailments were grouped, and then, the ICF values were calculated as:

$${\text{ICF}} = \frac{{n_{ur} - n_{t} }}{{n_{ur} - 1}}$$

Medicinal plants that were effective in treating groups of ailments had a higher informant consensus factor value.

The fidelity level (FL) computes the significance of a species for a given purpose. Most commonly used medicinal plants had high fidelity level value. The fidelity level (FL) among medicinal plants of the study area was computed based on the following formula: FL = Np/*N*. To calculate the percentage of fidelity level: FL% = (Np/*N*) × 100 was used [2]. Np is the number of informants who independently cited the importance of a species to treat a particular disease, and *N* is the total number of informants who reported the plant to treat any given disease.

The local importance of each species cited in the study area was calculated using use-value (UV) technique as follows [30]. Use-value (UV) is a quantitative method that demonstrates the relative importance of species known locally, which reflects the importance of each species to informants, i.e.,

$${\text{UVis }} = \, \Sigma U_{is} /n_{is}$$

where UVis = use-value of a species s for informant *i*, *U*_*is*_ = the number of uses mentioned in each event by informant *i*, and *n*_*is*_ = the number of events for species s with informant I.

## Results

### Medicinal plants diversity

A total of 98 medicinal plant species belonging to 87 genera and 48 families were reported to be used for treating human ailments in Suro Barguda District (Appendix 1). Family Fabaceae was represented by 10 species and followed by Lamiaceae (7 species). Four of the identified medicinal plants, i.e., *Bothriocline schimperi* Oliver & Hiern ex Bentham, *Erythrina brucei* Schweinf. emend. Gillett, *Lippia adoensis* Hochst. ex Walp. var. *adoensis,* and *Millettia ferruginea* (Hochst.) Hochst. ex Baker, which are all against human ailments were found endemic to Ethiopia. Identified growth forms of medicinal plants indicated that shrubs were represented by 36 species, trees by 30 species, and herbs by 14 species. Other forms such as lianas, climbing herbs, epiphytes, and succulents were represented with 11 species (11.2%), three species (3.1%), three species (3.1%), and one species (1.0%), respectively.

### Medicinal plant parts used

**Fig. 2:**
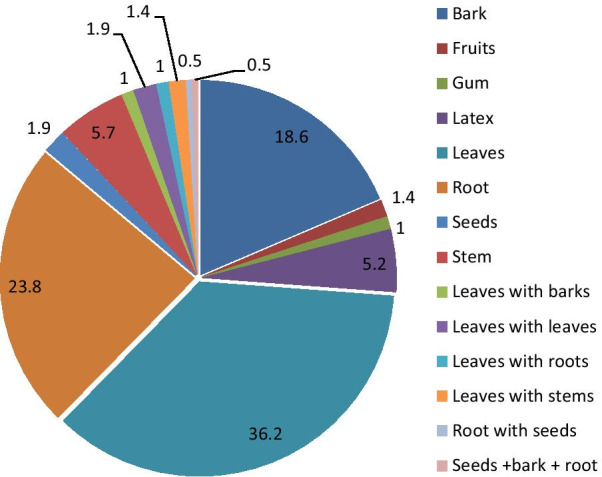
Parts of medicinal plants used for remedy preparation to treat human ailments in percent

Even though about eight different plant parts were reported to be used for remedy preparation in different ways, a larger proportion (36.2%) of the preparations were obtained from leaves followed by roots (23.8%) and barks (18.6%). In addition to this, the stem was used for 5.7% preparations, whereas the latex alone and the leaves in mixture with other plant parts were 5.2%, respectively (Fig. 2). Most of the remedy preparations (93.7%) were reported as they were prepared from freshly collected plant parts, 5.8% were prepared from dried parts, and the remaining (0.5%) were prepared either from the fresh or dried plant parts.

### Ways of herbal medicine preparation

**Fig. 3:**
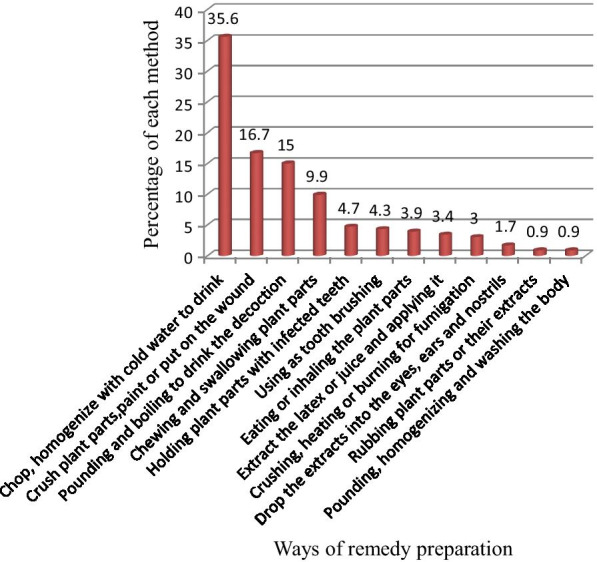
Ways of remedy preparation in treating human ailments in (%)

As herbalists reported in the study area, ways of preparing remedies vary based on the type of ailment which they identified based on symptoms observed on patients. The major way of herbal medicine preparation was through chopping or pounding the plant parts and homogenizing it with cold and clean water (35.6%) followed by crushing and put on the preparation (16.7%) and chopping, homogenizing, and boiling the preparation (15%) (Fig. 3).

### Route of remedy administration

Results of analysis of route of administration of medicinal preparations revealed that oral application was the most common route of administration (159 preparations, 70.4%) followed by dermal (49 preparations, 21.7%) and nasal administrations (12 preparations, 5.3%). Other remedies were reported to be administered through a deep opening on the body formed due to infection "Luxaa—in local Oromo language or tissue cancer" (three preparations, 1.3%); aural (two preparations, 0.9%); or ocular (one preparation, 0.4%) concerning the type of ailment reported by diagnosing the patient.

### Major types of diseases occurred in the study area with their clinical explanation

Out of the fifty-nine mentioned human diseases, thirty-one were considered for their traditional versus clinical explanations as indicated in Table 2.

**Table 2:** List of the top 31 most cited human health problems in the study area

Guji Oromo terms	Clinical terms	Clinical explanation
Albaatti	Diarrhea	Frequent bowel evacuation or the passage of abnormally soft, liquid feces
Baariille/Biiffaa/Roobbii	Ringworm	Fungal infection of the body, especially the face
Boocaa/Boowo	Severe head ache	Any disorder related to mental malfunctioning leading to loss of self-personality
Bookokaa/Dhukuba Garaa	Stomach ache	Any problems related to stomach either due to parasites, infections or allergy
Busaa	Malaria	Protozoal infection by anophilus Mosquito
Cacaa/Chiifee	Itch	Skin infection forming rashes which produce fluid and lastly becoming black in color
Cuma'a	Body disorder	Shivering and abnormal breathing
Danffaa Dhiiggaa	Hypertension	Loss of balance and head ache
Dhukuba Aaduu	Nerve disease	Disfunctioning of part of a body
Dhukuba Dhiraa	Gonorrhea	One of sexually transmitted disease
Dhukuba Gurra	Ear ache	Unpleasant feeling in the ear
Dhukuba iijaa	Eye ache	Eye infection due to different reasons
Dhukuba ilkaan	Tooth ache	Tooth infection
Dhukuba Muchaa	Breast cancer	Abnormal tissue growth in breast
Dhukuba Saree	Rabies	An acute virus disease of the central nervous system of all warm blooded animals
Dhukuba Sombaa	TB	Lung infection with mycobacterium tuberclosis causing caughing and apetite loss
Dhukuba Sukuarra	Diabetics	Unable to use blood sugar properly and need of energy giving food within short period
Dhulla	Body swelling	Swelling of part of the body due to infection
Gurroo/Shiinqaa	Asthma	Hypersensitive of the body to particular antigens in the breathing canal
Hadhaa Butti/Bofaa/Iddansaa Bofaa	Snake bite/venom	Infection with snake venom during its bite
Hadhoottuu	Increased bile production	Increased bile production due to malarial infection
Luxaa	Tissue cancer	Swelling and opening of hole in the tissue due to infection
Maagga	Ascaris	A disease caused by infestation of *Ascaris lumbricoides*
Madaa	Wound	Any infectious or mechanical injury to part of the body either with pus or dry
Megeenaa	Amoebiasis	An infection of the intestinal tract with Amoeba causing severe bloody diarrhea
Mujalee	Flee infection	Body, especially leg and hand, infection with Flee causing swelling and wound
Naqarsaa Garaa	Gastric ulcer	Inflammation of the lining of the stomach either acute or chronic stage
Qanxoo/Ciitto	Itching	Skin infection with Fungus causing severe itching
Qufaa/Yiikee	Coughing	Infection of breathing canal with virus or bacteria which cause coughing
Tiruu/Bekkekoo/Birttee/Tabbiisaa	Hepatitis	Liver disorders due to viral or bacterial infection
Waan Afaan	Tonsilitis	Inflammation of the tonsils due to bacteria or viral infection causing sore throat

### The most preferred medicinal plants for treating human ailments

Preference ranking exercise on medicinal plant species that were reported to be used against toothache, which was one of the gastrointestinal diseases common to the study area, showed the most effective medicinal plants (Table3).

**Table 3:** Results of preference ranking of ten medicinal plants reported for treating toothache

Medicinal plants used for toothache	Informants designated A to J										Total	Rank
	A	B	C	D	E	F	G	H	I	J		
*Acmella caulirhiza*	8	5	6	7	4	2	3	1	8	1	45	10th
*Capparis tomentosa*	8	9	7	6	4	5	3	8	1	2	53	7th
*Carissa spinarum*	8	9	7	10	6	4	5	3	1	7	60	3rd
*Clerodendrum myricoides*	10	8	9	7	10	10	3	1	10	2	70	1st
*Fagaropsis angolensis*	8	6	7	9	10	5	3	4	4	3	59	4th
*Pappea capensis*	7	6	5	8	4	8	3	1	3	2	47	9th
*Pittosporum viridiflorum*	8	7	9	6	4	5	9	1	4	3	56	5th
*Premna schimperi*	7	8	6	4	5	3	1	8	3	4	49	8th
*Rhoicissus revoilii*	9	7	6	8	5	4	2	9	3	1	54	6th
*Scherebra alata*	9	10	8	7	5	6	10	4	2	3	64	2nd

N.B. Scores in the table indicate ranks given to medicinal plants based on their efficacy. The highest number (10) was given for the medicinal plant which informants thought was most effective in treating toothache and the lowest number (1) for the least effective plant selected currently for this ranking purpose from numerous medicinal plants used to treat this disease

### Consensuses on the most frequently used medicinal plants used for treating human ailments in the study area

This study clarified that some medicinal plants were well known in the study area than others. Some medicinal plants and remedies prepared from them have cultural significance in all**-**around need (esthetic, historic, scientific, social, or spiritual) value among the Guji Oromo people at different times. As a result, all key informants cited such plants repeatedly as a remedy for various diseases of humans. For example, *Justicia schimperiana* (Hochst. ex Nees) T. Anders. and *Warburgia ugandensis* Sprague were cited by all key informants (100%) as sources of remedy for hepatitis and internal organ cancer, respectively. *Carissa spinarum* L., *Ocimum urticifolium* Roth.S.Lat. and *Schrebera alata* (Hochst.) Welw. were also cited by 23 (95.8%) key informants as sources of remedy for breast disease, febrile illness, and tooth cancer, respectively (Table 4).

**Table 4:** Key informant consensus on most commonly used medicinal plants

The botanical name of medicinal plants	Disease treated	No. of key informants	%
*Carissa spinarum*	Breast disease	23	95.8
*Clerodendrum myricoides*	Gonorrhea	22	91.7
*Haplocoelum foliolosum*	Parasitic worms	21	87.5
*Fagaropsis angolensis*	Coughing	20	83.3
*Ocimum urticifolium*	Febrile illness	23	95.8
*Justicia schimperiana*	Hepatitis	24	**100**
*Searsia pyroides*	Itching	19	79.2
*Schrebera alata*	Tooth cancer	23	95.8
*Warburgia ugandensis*	Internal organ cancer	24	**100**
*Withania somnifera*	Snake venom	20	83.3

Bold values indicate the medicinal plant spp. with higher informant consensus

### Effectiveness of medicinal plants

Informant consensus was used to evaluate the reliability of the data. To simplify the analysis, twelve disease categories were designated from the total 59 human ailments reported in the district and ICF values were computed based on the reported medicinal plant species and their use citations for each disease category, and the following results were obtained (Table 5).

**Table 5:** ICF values of traditional medicinal plants used for treating human ailments

Disease category	No. of spp.	% all spp.	Use citation	% all use citation	ICF
Dental, oral, and pharyngeal diseases	15	35.5	76	38.8	0.81
Dermatological diseases	14	21.1	136	69.4	0.90
Diabetics, hepatitis, and hypertension	12	15.8	39	19.9	0.71
Evil spirit diseases	7	9.2	24	12.2	0.73
External injuries and snake bite	13	30.3	72	36.7	0.83
Febrile diseases	5	6.6	58	29.6	0.90
Gastrointestinal diseases	16	6.6	194	99.0	**0.92**
Musculoskeletal and nervous system	13	56.6	68	34.7	0.82
Breathing system diseases	15	7.9	160	81.6	0.91
Sensorial diseases	13	17.1	42	21.4	0.70
Tissue cancer and cold disease	12	38.2	40	20.4	0.72
Urogenital and venereal diseases	9	25.0	33	16.8	0.75

Bold values indicate ICF with a higher value

### The relative healing potential of medicinal plants used for treating human ailments

Fidelity level values of medicinal plants commonly reported against a given human ailment category were computed to know the healing potential of the reported medicinal plants against the corresponding diseases and the following results were obtained (Table 6).

**Table 6:** Fidelity level values of medicinal plants commonly reported against a given human ailment category

Medicinal plant	Therapeutic category	Np	*N*	FL (%)
*Combretum molle*	Gastrointestinal parasites	28	29	**97**
*Fagaropsis angolensis*	Breathing system diseases	23	24	96
*Ocimum urticifolium*	Febrile diseases	14	15	93
*Momordica foetida*	Rabies and Gonorrhea	11	12	92
*Pappea capensis*	External injuries and snake bite	16	19	84
*Rhus vulgaris*	Dermatological diseases	18	19	95
*Schrebera alata*	Dental, oral, and pharyngeal	13	15	87
*Solanum incanum*	Hepatitis and stomach ache	17	20	85
*Warburgia ugandensis*	Tissue cancer and cold disease	12	14	85
*Withania somnifera*	Evil spirit diseases	10	12	83

Bold values indicate medicinal plant species with a higher FL value

*FL* fidelity level, *Np* number of informants who independently cited the importance of a species for treating a particular disease, *N* total number of informants who reported the plant for any given disease

### Medicinal use-values of selected plant species

Traditional medicinal uses of different medicinal plant species against several human ailments were compiled. Investigation of their use-value indicated that some of these plants show high medicinal use-value (UVmed) as indicated in Table 7.

**Table 7:** Medicinal use-values (UVmed) of most cited human medicinal plants in Suro Barguda District

Medicinal plant species	No. of informants citing the species	Total citations	No. of ailments treated with	UVmed.
*Asparagus africanus*	81	605	4	7.4
*Capparis tomentosa*	118	980	5	8.3
*Cucumis pustulatus*	92	589	5	6.4
*Euclea divinorum*	60	402	4	6.7
*Ehretia cymosa*	120	912	7	7.6
*Pappea capensis*	140	1232	8	8.8
*Premna schimperi*	104	676	6	6.5

### Identification of multipurpose medicinal plants for their conservation priority

The result of the direct matrix ranking exercise on the selected medicinal plants used for treating human ailments enabled us to identify which of the multipurpose plants was under greater pressure than other species in the area beside the respective factors that threaten the plants (Table 8).

**Table 8:** Average direct matrix ranking score of ten key informants for ten medicinal plant species with additional uses

Medicinal plant species	Use categories					Total	Rank
	Ch	Co	Fr &Tl	Fw	Md		
*Allophylus abyssinicus*	5	3	2	3	2	15	4th
*Combretum molle*	1	3	3	4	3	14	5th
*Ehretia cymosa*	2	1	2	3	1	9	10th
*Fagaropsis angolensis*	3	3	4	5	3	18	2nd
*Olea europaea* subsp. *cuspidata*	3	5	2	4	2	16	3rd
*Pappea capensis*	3	3	2	2	1	11	8th
*Podocarpus falcatus*	1	2	1	4	2	10	9th
*Schrebera alata*	1	3	3	3	2	12	7th
*Terminalia brownii*	3	2	2	4	2	13	6th
*Warburgia ugandensis*	4	5	4	3	4	20	1st
Total	26	30	25	35	22	138	
Rank	3rd	2nd	4th	1st	5th		

N.B. Scores in the table indicate ranks given to medicinal plants based on their use diversity. The highest number (5) was given for the medicinal plant which informants thought was used commonly for the mentioned purpose and the lowest number (1) for the least needed for that purpose

*Ch* charcoal, *Co* construction, Fr & Tl furniture and tools, *Fw* firewood and *Md* medicinal

### Use diversity of medicinal plants

**Fig. 4:**
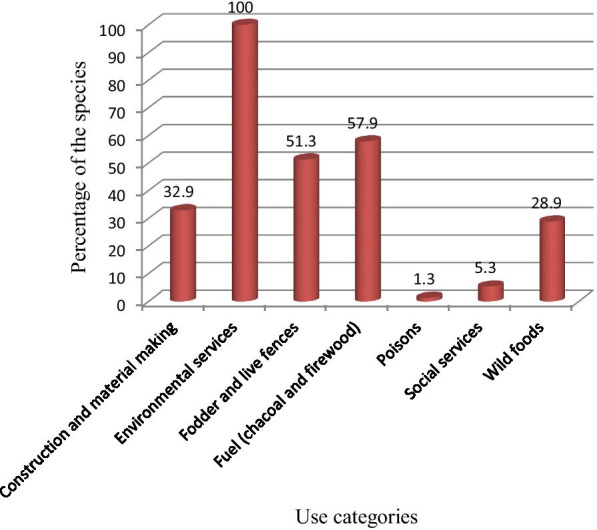
The proportion of human medicinal plants over different use categories

All the 98 medicinal plant species recorded for treating human ailments in the district were cited for one or more uses other than their medicinal role. The proportion of medicinal plant species over different use categories is summarized in Fig. 4.

### Solvent and additives used in traditional herbal medicines prepared in the study area

**Fig. 5:**
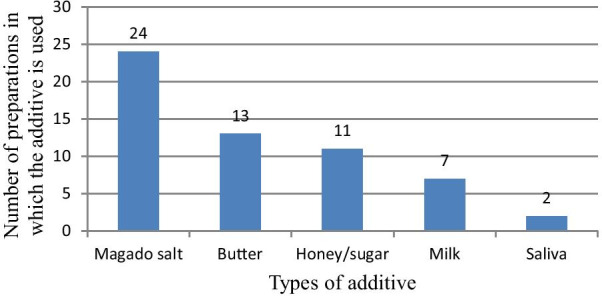
Additives used in the preparation of traditional remedies

Almost in all ethnoformulations of traditional medicines, water was reported to serve as “solvent” whenever dilution is required. Different additives were incorporated in 23.4% of the whole ethnoformulations, and “Magado” salt (locally produced salt) was the most commonly used additive (Fig. 5).

### Distribution of indigenous knowledge on medicinal plants among different social groups in the community of the study area

The difference in naming medicinal plants between different social groups (elders, youngsters, men, women, literate, illiterate, key informants, randomly taken informants, etc.) was analyzed by using a statistical test as follows (Table 9).

**Table 9:** Statistical test of significance on the average number of medicinal plants among different informant groups in Suro Barguda District

Considerations	Informant groups	*N*	Average ± SD	*t* value**	*p* value
Gender	Men	168	6.62 ± 2.65	1.97	*p* ≤ 0.97
	Women	28	6.05 ± 2.18		
Age	Youngsters (< 40 years old)	112	5.16 ± 2.07	− 12.87	*p* ≤ 0.001*
	Elders (> 40 years old)	84	7.96 ± 2.19		
Literacy	Illiterates’ participants	122	7.22 ± 2.31	12.92	*p* ≤ 0.001*
	Literates’ participants	74	4.28 ± 1.82		
Proximity to the health center	Near to health center	32	6.00 ± 2.37	− 0.94	*p* ≤ 0.36
	Far away from the health center	164	6.5 ± 2.57		
Informant category	Key informants	24	10.76 ± 1.09	25.75	*p* ≤ 0.001*
	Randomly taken informants	172	5.85 ± 2.04		

Degree of freedom (*df*) = 223

*N* = number of respondents

*Significant difference (*p* < 0.05)

**t(0.05) (two-tailed)

### The market survey of medicinal plants

A market survey was done in the biggest market of the district (Suro Market) to observe and collect data on the marketability and trade of medicinal plants. The researchers observed as some medicinal plants were sold for other purposes than their reported medicinal uses.

## Discussion

In Suro Barguda District, a large number of medicinal plants (98 medicinal plant species) were reported which were used to treat human ailments and the finding indicated the presence of considerable medicinal plant diversity. Fabaceae contributed 10 species for remedy preparation in treating different human affecting diseases. As indicated in [31–33], this could be due to the dominant presence of Fabaceae, Asteraceae, and Lamiaceae families in the flora of Ethiopia and Eritrea. The existence and utilization of such a large number of medicinal plants might indicate that the majority of the people continue to employ indigenous medicinal practices to date. Four of the medicinal plants, *Bothriocline schimperi, Erythrina brucei*, *Lippia adoensis* var. *adoensis,* and *Millettia ferruginea*, were endemic to Ethiopia. *Bothriocline schimperi* was used to treat severe headaches, *Erythrina brucei* was used to treat toothache, *Lippia adoensis* was used to treat body swelling and *Millettia ferruginea* was used to treat cold and flee infection. This indicates that the endemic flora of Ethiopia deliver diverse medicinal uses*.* Identified growth forms of medicinal plants indicated that shrubs were more dominant (represented by 36 species), a report which is in agreement with other studies in Ethiopia such as [34–39] who reported dominance of shrubs in their medicinal flora. This could be due to the high occurrence and impact tolerance capacity of shrubs in the study area. Almost all of the medicinal plants were obtained from the wild. But, the wild habitats were highly depleted due to an increased human and livestock population which resulted in the loss of many medicinal plant species growing in the wild. This finding was in agreement with reports of almost all other studies on wild habitats for harvesting an ample of medicinal plants such as [40–43] which clarified that most medicinal plants are adapted well and available plenty in wild areas but highly affected due to their overexploitation for different purposes.

### Parts of medicinal plants used for remedy preparation

A variety of plant parts were reported as to be used for remedy preparation in the district, but about 36.2% of the preparations were obtained from leaves which was also reported by other ethnomedicinal studies elsewhere in Ethiopia [39, 41, 44–46], which mentioned the maximum percentage of the species were harvested for their leaves followed by roots (23.8%) and barks (18.6%). In addition to this, the leaves, barks, and roots were used in different preparations in mixture with other plant parts in the district. The higher usage of leaves in traditional remedy preparations could partly be due to its easy availability and the usage of roots in the second place could be because these parts remain in the soil which enables them to be taken at any time even during the long dry season, especially in arid and semiarid areas like the present study area. Assumed the highest frequency of leaves used for medicinal purposes in the study area threat to the destruction of medicinal plants, especially to trees and shrubs, was found to be a minimal, as high threat to the mother plant comes with root, bark, and leafy stem harvests. Roots affect the survival of the plant since aerial parts of the plant are highly dependent on it for physiological processes and physical support as these parts are the second most frequently used parts in the study area. However, Cunningham [47] indicated that the harvest of leaves has also a threat to the deterioration of medicinal plants since the removal of leaves limits the transformation of vegetative to reproductive development such as flower production and fruit/seed set, which in turn limits the natural/wild regeneration of plants. According to [48], medicinal plant harvests that mainly involve roots, stems, and barks have a serious effect on the survival of mother plants. Most of the remedy preparation was reported from freshly collected plant parts (93.7%). Using freshly harvested medicinal plant parts could be due to the belief that this form could attain high efficacy since it could contain higher bioactive ingredients (curative elements, a knowledge which could be obtained from long and repeated experience). Sofowara [49] had declared that the uses of fresh medicinal plants were more effective than other parts. The reliance of local people on fresh materials in the study area including the usage of fresh barks and leaves put the plants under serious threat than the dried form, as fresh materials are collected directly and used soon with its extra deterioration with no chance of preservation, i.e., not stored for later use. This finding was also in line with other ethnomedicinal studies elsewhere in the country [40, 46, 48, 50] which indicated that most of the remedies were prepared/processed from fresh plant parts, whereas dried parts were used least and a certain amount of remedies was reported to be prepared from both dried or fresh parts of medicinal plant species.

### Types of disease, medicine preparations, and treatment methods

Fifty-nine disease types, affecting humans, were identified in Suro Barguda District to be treated with traditional medicines (Additional file 1). Out of the major disease categories in the study area, gastrointestinal diseases such as toothache, stomach ache, and diarrhea were the most frequently reported human ailments mainly treated traditionally using a large number of medicinal plants. This may be due to the spreading of various pathogens as a result of less hygiene and control measures in such rural areas. This could also validate the effort of local healers in probing out more and suitable medicinal plant species for the treatment of such diseases. Similar research findings were reported by Abera [51] which disclosed that of the major disease categories in that study area, infectious diseases are mainly treated traditionally using a large number of medicinal plants. This may be due to the distribution of various pathogens as a result of less sanitation and control measures in developing countries. These diseases were diagnosed commonly through interviews and visual inspection of the patients before any herbal medicine administration. Similar reports were given by [40, 52, 53] who stated that diseases were diagnosed commonly through interviews and visual inspection of the patients, and dosages were determined according to the age, sex, and physical appearance of the patient. Once the healer gets the required information, herbal medicines would be prepared and administered following the proper route and the type of disease. The major mode of herbal medicine preparation for human ailments was chopping/crushing or pounding and homogenizing it with cold water, and findings which were similar to the reports of [46] indicated that very dominant methods of remedy preparation were reported to be through crushing (grinding). This was followed by crushing and put on plant parts and chopping, homogenizing, and boiling plant parts. The oral application was the most common route of administration (70.4%), a report similar to [40, 43] which clarified that oral application of remedies was popular followed by dermal and nasal administrations. Other remedies were reported to be administered through a deep opening on the body formed due to infection “Luxaa—in local Guji Oromo language,” aural and ocular routes concerning the type of ailment reported during diagnosing the patient. It was reported that determination of remedy dose for various ailments was based on physical appearance, age, and gender of the patient (no standardized measurements) except determining it based on the long-term experiences of traditional healers. Similar findings were reported in other parts of the country [50, 54] which mentioned that the dosage prescription for children was mostly lower than for adults. Remedies were reported to be measured in coffee cups, water glasses, liters while others were measured with the tip of fingers or pieces of particles, a finding similar to the report of [50] which indicated that dosages were estimated using lids, spoons, cups, glasses, pinches or handfuls. As mentioned by Friis et al. [20], lack of standardization and precision has been a global shortcoming of the traditional healthcare system. Additives in traditional medicine preparations are commonly used for various purposes including an increase in the healing potential of the remedy, improving flavor and taste and also to avoid abdominal discomfort. Milk, yogurt, honey, and coffee were mentioned as antidotes for traditional medicines for adverse side effects such as vomiting, diarrhea, and feeling of burning and sometimes weakening of the patient. Similar reports were forwarded by [41, 55, 56] who reported remedies are mixed with additives to improve flavor and healing potential in their respective study sites. Patients with gastrointestinal problems, venereal diseases, malaria, hepatitis, hypertension, diabetics, TB, rabies, poisons, etc. were commonly reported to be treated with liquid preparations or chewable plant parts given orally. This finding was in agreement with reports of [54] which clarified that the highest numbers of plant species were reported to be used for the treatment of abdominal/stomach disorders and internal parasites. Those with different skin diseases and tissue cancer were reported to be treated with crushed or chewed preparations through rubbing or pasting herbal preparations. Diseases such as febrile illness, headache, and evil eye were reported to be treated either through fumigation or washing the patient with liquid herbal preparations. Culture, efficacy, availability, and economic factors were reported as the key factors which lead the community to use traditional medicines other than the unaffordable and non-available modern healthcare services in the area. These findings were in agreement with the findings of [40, 57] which stated that economic, cultural, efficacy, and availability factors were reported as the key factors which lead the community to knock at the door of traditional healthcare practitioners than the few distantly located healthcare centers with unaffordable prices.

### The most preferred plants for treating human ailments

The output of preference ranking exercise on medicinal plants that were reported to be used against toothache showed that *Clerodendrum myricoides* (Hochst.) Vatke were the most preferred species followed by *Scherebra alata* (Hochst) Welw and *Carissa spinarum* L. This indicated that indigenous people of the study area had sufficient knowledge of the healing potential of medicinal plants for different diseases. Consensuses on most frequently used medicinal plants indicated that *Justicia schimperiana* (Hochst. ex Nees) T. Anders. and *Warburgia ugandensis* Sprague were well-known medicinal plants in the study area in treating hepatitis and internal organ cancer, respectively. Informant consensus means agreement among informants. Selecting traditional medicinal plants by using informant consensus was used to evaluate the reliability of the data. Among the twelve categorized human ailments, the highest informant consensus factor value (ICF value) indicated that breathing system diseases were the most frequently occurring diseases. Mwambazi [7] expressed that high ICF values are important to name plants of particular interest in the search for bioactive compounds. The highest plant use citation was seen for gastrointestinal diseases. The observed high informant agreement together with high plant use citations for these disease categories could also indicate the relatively high occurrence of the diseases in the area. (About 16 medicinal plant species were cited for treating gastrointestinal disease categories.) Culture can demonstrate the way a group thinks, their practices, or behavioral patterns, or their views of the world. Therefore, traditional medicinal plants and remedies prepared from them have cultural significance in an esthetic, historic, scientific, social, or spiritual value among the Guji Oromo people for past, present, or future generations. Esthetic values of medicinal plants are those values that determine what people perceive as good to the content, integrity, harmony, and purity of medicinal plants and remedies prepared from them. Medicinal plants, also called medicinal herbs, have been discovered and used in traditional medicine practices since prehistoric times which indicate their historic value. Revitalization and renewed interest in traditional medicinal plants have been observed among the public and scientific community refers to their scientific value [58]. The role, contributions, and usefulness of medicinal plants in tackling the diseases of public health clarify its social importance. Illness is regarded as having both natural and supernatural causes and thus must be treated by both physical and spiritual means, using divination, incantations, animal sacrifice, exorcism, and herbs that refer to their spiritual values.

### The relative healing potential of medicinal plants used for treating human ailments

The highest fidelity level value was recorded for *Combretum molle* R.Br. ex G.Don in treating the gastrointestinal disease therapeutic category which was followed by *Fagaropsis angolensis* (Engl.) Dale used to treat breathing system diseases. These values could be a clue for the high healing potential of these plants against the corresponding diseases. An informant consensus factor (ICF) value was used to evaluate the reliability of the data, and these values were computed based on the reported medicinal plant species and their use citations. Lulekal et al. [59] stated that plants scoring higher informant consensus values are thought to have better potency containing biologically active ingredients in treatment as compared to plants with less informant consensus values. In this analysis, the use citation for medicinal plants used to treat gastrointestinal diseases was higher (92%) and informant consensus factor values (ICF values) were used to identify the harmony of the informants on the reported cure for the group of ailments (gastrointestinal diseases) of the plant while fidelity level (FL) computes the significance of a species (*Combretum molle*) to treat a given disease (gastrointestinal diseases). Hence, their analysis values inveterate as the information obtained was real.

### Use diversity of medicinal plants

Medicinal plant species recorded for the treatment of human ailments in the district were cited for one or more uses other than their medicinal role. All medicinal plant species were considered useful for environmental services such as control of erosion, soil improvement, being food and shelter for wild animals, and balancing climatic conditions which is the general truth with no compromise. The dominance of plants cited for environmental services was proper from the standpoint that every plant species has its role in maintaining balanced biophysical systems. About 55 woody medicinal plant species of the district were used as fuel (charcoal and firewood) for the local people, and 48 medicinal plant species were cited for additional uses as fodder indicating their supplementary role in supporting the livestock wealth of the district, on which most people depend for their livelihoods. About 30 medicinal plant species were also reported for construction and material making, 22 medicinal plant species were cited as wild edible plants, five medicinal plant species were indicated as plants giving social services, and two medicinal plant species were reported as being poisonous. These results indicated how much the indigenous knowledge of the local people was used in using plant resources for different purposes to ensure their existence besides fulfilling their requirements. On the other hand, the highest proportion of plant species used across different use categories reflects the relative importance of different plant species in people's daily life and a similar notion was reported by Federal Democratic Republic of Ethiopia [60] who mentioned that different plant species were identified as to have different use categories.

### Conservation priority of multipurpose medicinal plants

Ethiopia is one of the countries which signed the Convention on Biological Diversity (CBD) on June 10, 1992, and ratified on April 5, 1994, by ratification (Proclamation No. 98/1986) [61]. The conservation and sustainable use of biodiversity are critically important to the aspiration and policies of the health sector—helping to secure the basic human right to health and supporting universal primary health care. Biodiversity loss and ecosystem degradation pose a growing threat to our health and well-being, but protecting biodiversity can help to strengthen public health programs, reduce emerging health risks and sustain resources for medicine and medical research. Similarly, achieving sustainable health for all people is important for conservation and the long-term success of biodiversity strategies and action plans.

Disease and the lack of access to healthcare services place significant pressures on community resources, promoting unsustainable exploitation of biodiversity. The health sector also has its impacts on biodiversity, and health interventions, particularly in emergencies, have the potential for ecological damage. It is increasingly important for the public health sector to recognize that human health and well-being are influenced by the health and integrity of local ecosystems, and frequently by the status of local plant and animal communities. In many cases, the long-term success and sustainability of public health management planning may be determined by the degree to which ecological factors are taken into account.

Cooperation between the health and biodiversity sectors is essential if these linkages are to be addressed effectively for the long-term benefit of all life. Conservation and sustainable use of biodiversity must be recognized as an important element of public health management planning. Appropriate steps for safeguarding ecosystem services must be incorporated into national action plans for promoting health and delivering universal primary health care. This should include mainstreaming biodiversity into policies and plans relating to the health impacts of climate change. It is also important to ensure that the potential impacts of current health and development activities on biodiversity are understood and addressed so that possible future risks can be avoided. So, sustainable development requires a holistic approach that acknowledges the links between biodiversity and human well-being [62].

The indigenous religion of Guji Oromo (*Waaqeffannaa*) has a positive contribution to natural resource management. In this indigenous belief, cutting big trees is out of bounds, because when it falls it has a very huge sound and power which would disturb God (*Waaqa*) and all humans closer to the area as well. Big trees are respected like big elders in Guji Oromo people. Forests have appealing value and certain early ancestral historical bonds with the community. Due to disturbed climatic conditions, minimized agricultural production, and economic crisis, the care for natural resources is minimizing nowadays. Several studies [52, 63, 64] also indicated that an increase in human and livestock population, as well as growing investment in agriculture, became the major threat for the deterioration of vegetation in general and medicinal plants in particular. The study by Bekele [64] also indicated that the use of firewood and construction, as well as agricultural expansion, were the main causes for the diminution of medicinal plants in the study area. Direct matrix ranking exercise for ten selected multipurpose medicinal plants in five use diversities (charcoal, construction, furniture, and tools, firewood, and medicinal) indicated that *Warburgia ugandensis* was ranked first (most threatened) which was followed by *Fagaropsis angolensis* and *Olea europaea* subsp. *cuspidata* (Wall. ex G.Don) Cif. L’Olivicoltore. Since stem bark of *Warburgia ugandensis* was highly and unwisely used in treating different diseases in Guji Oromo traditional medication, its population was reaching nearly zero (lost through drying) with the effect of disturbances (grazing, browsing, and selective cutting) which seriously affect the emergence of seedlings and their growth to saplings. In the same way, poor reproduction ability, selective cutting mostly for construction purposes, overgrazing, and browsing affect the existence of *Fagaropsis angolensis*. Even if *Olea europaea* subsp. *cuspidata* has good reproduction ability, stable population structure and good regeneration status, cutting of bigger individuals for firewood and the destruction of seedlings and saplings due to overgrazing and browsing has affected the species. Therefore, the output of this study indicated that these multipurpose medicinal plant species were currently exploited more for their non-medicinal uses such as for firewood, construction, and charcoal production. Hence, these findings reflected the requirement of an urgent harmonizing conservation action to save the fast-eroding multipurpose medicinal plant species of the study area. Fabricant and Farnsworth [58] had also reported the same pattern of high exploitation of multipurpose medicinal plants.

### Distribution of indigenous knowledge on medicinal plants among different social groups in the community of the study area

Although more medicinal plants were reported by men (168) than women (28), the difference was not significant (*p* > 0.05) when the average number of medicinal plants mentioned by each group was compared. There was no significant difference seen in the number of medicinal plants listed by informants living around health centers and those living relatively far away from these health centers. However, there was a significant difference (*p* < 0.05) in the number of medicinal plants reported by senior members of the community (> 40 years old) and young- to middle-aged members (< 40 years old): key informants and randomly taken informants, illiterates’ and literates’ participant (Table 9). More number of medicinal plants was reported by elders (> 40 years old), illiterates, and key informants than by young, literates, and randomly taken informants. Deteriorating a positive attitude toward the use of traditional medicine by young generations indicates the loss of the main indigenous knowledge. Simbo [65] in Cameroon indicated in his study that most young people in urban areas were not interested in the use of traditional medicine due to the effect of Western culture, the manner in mind that traditional medicine is credulous, which is mainly used by deprived and illiterate people. Alternatively, most of the elders kept their knowledge secret in thinking of the medicine become ineffective if it is told to everybody. Likewise, the decline of the traditional knowledge in the generation is due to the intrusion of and shifts to the use of more artificial drugs not only in the urban but also extending to the countryside. Studies [66, 67] indicated that the lack of formal education in traditional knowledge in developing nations is another factor for the weakening of indigenous knowledge because there was no way of documenting and transfer it to the next generation. Furthermore, most of the African contemporary health professionals significantly emasculate the contribution of traditional medicine in the healthcare system; however, the scientists of developed nations exhaustively search for medicinal plants to search for a solution for the hoary and newly rising diseases, especially viral diseases. All these factors may result in a loss of this precious and useful knowledge that has been accumulated over many generations.

### Marketability of medicinal plants

The finding from the market survey of medicinal plants indicated that in the culture of Guji Oromo (an ethnic group of the study area) it is forbidden to sell traditional medicine in the market. Even if people coming from other areas usually sell them, the local people did not accept it. When traditional healers cure a patient, they will be compensated by giving some things in kinds such as a certain amount of dried and pounded tobacco, coffee with salt or sugar, and if it is a critical disease a goat or heifer not money as the cultural rule permits. Sometimes the cured person returns the favor by inviting the healer and his family overnight in his house. This cultural trend might cause the local healers, not to sell traditional medicines in the market which was confirmed by the market survey (Suro Market). But some traditional medicinal plants can be sold for other purposes such as *Combretum molle*, *Olea europaea* subsp. *cuspidata*, and *Osyris quadripartita* Decn. for fumigation in producing good odor and feeling for the body, milk containers, and the house.

## Conclusion

The study area was rich in having medicinal plants and corresponding indigenous knowledge diversity. Findings of this study indicated that there was higher usage of leaves of most medicinal plants to prepare various traditional remedies. Even though collecting these parts seems not harming much the regular physiological activities of the plant, those plants only with a limited number of leaves can be endangered unless proper consideration is given. Hence, conservation work in the area needs to give prior attention to protect such types of plant species. Traditional practitioners diagnose their patients through observation and asking the patient about the feelings of the disease and then prepare the medicine to administer it accordingly based on their cultural knowledge on symptoms, corresponding illnesses, and therapeutic medicinal species held in the knowledge of indigenous people. This would have been more effective if these people have obtained certain training from modern health professionals about how to identify some diseases based on their symptoms, especially those which are easily communicable to minimize the possible severe problems that could happen in the local community and how to determine the doses of the preparations.

Traditional practitioners showed varying degrees of traditional medicinal plant use knowledge based on differences in age, experience, gender, and education level. More ethnomedicinal knowledge was observed in elderly members of the community than in younger groups; experienced/key practitioners than the general public; and more with the illiterate than the literate. Because of the cultural norm and secrecy of the traditional medication system, this knowledge was transmitted along the selected male line of the family members due to which males could be more knowledgeable than females even if the difference in knowledge concerning gender was not exaggerated in the study area. Oral transmission of traditional knowledge and its flow only through the selected male line of the family for its secrecy may cause it to be depleted beside impacts of modernization and ignorance of the new generation. High ICF, FL, and medicinal use-values testing exercises result showed that the selected medicinal plants of Suro Barguda District had promising bioactive elements.

## Supplementary Information

**Additional file 1**. Summary of medicinal plants used to treat human ailments in Suro Barguda District.

## Data Availability

All data supporting our finding are attached as Additional file 1.
