# Supplementary material for: Cultural significance of medicinal plants in healing human ailments among Guji semi-pastoralist people, Suro Barguda District, Ethiopia
Source: J Ethnobiol Ethnomed. 2021 Oct 18;17:61. doi: 10.1186/s13002-021-00487-4 (PMC8524801; doi:10.1186/s13002-021-00487-4)
Supplement: Supplementary file 1 — Additional file 1. Summary of medicinal plants used to treat human ailments in Suro Barguda District. [file 13002_2021_487_MOESM1_ESM.docx]

**Cultural significance of medicinal plants in treating different human ailments in Guji Semi-Pastoralist People, Suro Barguda District, West Guji Zone, Oromia Regional State, Ethiopia.**

Authors name: ^1^Mersha Ashagre Eshete (PhD); ^2^Ermias Lulekal Molla (PhD)

Address: 1Department of Biology, College of Natural and Computational Sciences, Bule Hora University, P.O. Box 144, Cell Phone +251930528889 or +251913247601, E-mail: mae19590917@gmail.com; 2Department of Plant Biology and Biodiversity Management, College of Natural and Computational Sciences, Addis Ababa University, P.O.Box 34731, E-mail: Zeaklog@gmail.com

**Additional file:** Summary of medicinal plants used to treat human ailments in Suro Barguda District; Key: (Hb=Habit, Pu=Parts used, Ut=Used to treat, Cp=Condition of preparation, Ra= Route of application, T=Tree, H=Herb, Sh=Shrub, Cl=Climber, Ls=Livestock, F=Fresh, D=Dried, F/D=Fresh/Dried, O=Oral, Dm=Dermal, Na=Nasal, Op=Optical, Er=Ear, L=Leaf, Rt=Root, St=Stem, Ba=Bark, Fl=Flower, Fr=Fruit, S=Seed, Bu=Bulb, Rh= Rhizome, La=Latex, Ds=Distribution, C=Common, R=Rare, *=Endemic).

**Appendix I. Medicinal plants used to treat human ailments in Suro Barguda District**

**Key**: (Hb=Habit, Pu=Parts used, Ut=Used to treat, Cp=Condition of preparation, Ra= Route of application, T=Tree, H=Herb, Sh=Shrub, Cl=Climber, Hu= Human, F=Fresh, D=Dried, F/D=Fresh/Dried, O=Oral, Dm=Dermal, Na=Nasal, Op=Optical, Er=Ear, L=Leaf, Rt=Root, St=Stem, Ba=Bark, Fl=Flower, Fr=Fruit, S=Seed, Bu=Bulb, Rh= Rhizome, La=Latex, Ds=Distribution, C=Common, R=Rare, *=Endemic).

| **No.** | **Scientific name** | **Family** | **Local name**  **(Oromo language)** | **Hb** | **Pu** | **Ut** | **Cp** | **Ra** | **Disease treated** | **Preparation & Application** | **Ds** | **Vouch. No.** | | | |  |
| --- | --- | --- | --- | --- | --- | --- | --- | --- | --- | --- | --- | --- | --- | --- | --- | --- |
| 1 | *Acacia brevispica* Harms | Fabaceae | HAMARROO | T | R | Hu | F | Dm  & O | Swelling &  forming  wound on  the body  (LUXAA) | The root is crushed, some of it is boiled as tea, and 1 coffee cup is given for the patient. Some of the crushed root is put on the opening of the wound. | C | MA45 b | | | |  |
| 2 | *Acmella caulirhiza* Del. | Asteraceae | JILLOO QALDHAA | H | L & R | Hu | F | O | Abdominal ache (GARAA BU’E) , Cold disease  (GAMTOKKE) and breast cancer.  Inflammation of  children’s  mouth  (WAAN AFAAN)  Tissue Cancer (LUXAA)  Tooth ache (DHUKKUBBII ILKKAAN) | Chopping the leaves, making s/n and giving one tea cup once per day for the patient.  The root is crushed, boiled as tea and 1 coffee cup is given to the breast cancer patient.  Chopping the leaves, making s/n and giving ½ of coffee cup.  Chopping the leaves, making s/n, inserting the s/n through the opening and closing the mouth of the opening with the residue.  Chopping the leaves and holding with the infected tooth. | C | MA14 | | | |  |
| 3 | *Acokanthera schimperi* (A.DC) Schweinf | Apocyanaceae | QARAARRU | T | L | Hu | F | Dm | Itching (QANXOO/  CIITTO) | Crushing the leaves and applying on the affected part. | C | MA63 | | | |  |
| 4 | *Albizia schimperiana* Oliv. | Fabaceae | GARBII | T | Inner part of bark | Hu | F | O | Stomach ache  Stomach cancer | Crushing the bark, making s/n and giving 1 water glass per day for 3 days.  Crushing internal bark of this plant with that of *Ekebergia capensis* bark , making s/n and giving one water glass 2 times a day for one day. | R | MA09 | | |  |  |
| 5 | *Allophylus abyssinicus* (Hochst.) Rodlkofer | Sapindaceae | SARAJII | T | Fr | Hu | F | O | Stomach ache  (DHUKKUBA GARAA) | Eating the fruit. | R | MA302 | | |  |  |
| 6 | *Aloe trichosantha* Berger | Asphodelaceae | HARGISSA | Suc | L & Lat | Hu | F and heated | Dm  O | Skin cancer and wound.  Skin fungi (Ring Worm)- (BARRILLE)  Hepatitis (BIIRTEE) and  Increased bile production due to malarial infection (HADHOOTTUU)  Eye disease  Malaria (BUSAA) | Cutting and chopping the leaf or peeling the leaf and applying on the affected part.  Applying the latex on the infected part.  Crushing the leaves with magado salt and boiling it with water and giving 1 coffee cup once for the patient.  Dropping one drop of its s/n in to the infected eye 2 times a day for 3 days.  Taking the latex about half of coffee cup adding some water and drinking at once. | R | MA280 | | |  |  |
| 7 | *Asparagus africanus* Lam. | Asparagaceae | HIDDOO | Li | L | Hu | F | O | Swelling of the breast due to cold disease  (GAMTOKKE) | Crushing the leaves, making s/n, adding honey and giving one water glass for the patient 2 times a day for 3 days. | C | MA25 b | | |  |  |
| 8 | *Asparagus flagellaris* (Kunth) Baker | Asparagaceae | SARRITTI | Li | L | Hu | F | O | Increased bile production due to malarial infection (HADHOOTTUU).  Wound (MADAA) and swelling of part of the body due to infection (DHULLAA). | Crushing the leaves with megado salt and giving half of a coffee cup of the solution to the patient once.  Crushing the leaves with the leaves of *Cadaba ruspolii* and magado salt to apply on the wound or giving half of coffee cup of its s/n once per day for swelling. | C | MA17 | |  |  |  |
| 9 | *Balanites aegyptiaca* (L.) Del. | Balanitaceae | BADANAA | T | R & Fru.  Gum (Hamphee) | Hu | F | O | Tooth and stomach ache.  Gonorrhea  (DHUKKUBA  DHIIRAA).  Breast cancer (NAQARSSA MUCHA ) | Piece of root is chewed for tooth ache and the fruit is eaten for stomach ache.  Crushing the root, making s/n and giving one water glass 2 times a day for 3 days.  Chewing the gum.  Crushing the root, making s/n and giving one coffee cup. | C | MA23 | |  |  |  |
| 10 | *Balanites rotundifolia* (van Tieghem) Blatter | Balanitaceae | BADANA OKOLEE | T | St,  Gu | Hu | Dr | Fum | Head ache (BOWO).  Gonorrhea  (DHUKKUBA  DHIIRAA) and Amoeboid (MEGEENAA) | Taking dried parts of the stem fumigating the patient for head ache.  Crushing the gum, boiling it with butter and giving 1 coffee cup 2 times/day. | C | MA93 | |  |  |  |
| 11 | *Bersama abyssinica* Fresen. | Melianthaceae | XIIBIRROO | T | L | Hu | F | O | Amoeboid (MAGEENAA) and Ascaris  (MAAGAA) | Chopping young leaves, making s/n and giving 1 water glass 2 times for 3 days. | C | MA152 | |  |  |  |
| 12 | *Bothriocline schimperi** Oliv. & Hiern ex Benth. | Asteraceae | FAKKATA ANCABII | Sh | L | Hu | F | O | Severe headache (BOCAA) | Chopping the leaves, squeezing by adding some water, drinking ½ of a coffee cup, and dropping 1 - 2 drops through the nose. | C | MA 01 | |  |  |  |
| 13 | *Cadaba ruspolii* Gilg | Capparidaceae | SAPHANSA | Sh | Ba | Hu | F | O | Hepatitis | Its bark is chopped and boiled with megado salt and 3 - 4 coffee cup solution is given to adult human patient/day. | R | MA59 | |  |  |  |
| 14 | *Calpurnia aurea (Alti)* Benth. | Fabaceae | CEEKATTA | Sh | L | Hu | F | O  Er | Hepatitis (BIIRTEE)  Ear ache (DHUKKUBI  GURRA)  Hypertension  (Human) | Chopping the leaves with magado salt, making s/n and giving one coffee cup for the patient.  Crushing the leaves, making s/n, filtering and dropping 2 drops into the infected ear 2 times a day for 3 days.  Toasting 7 seeds, crushing, adding to a cup of boiled coffee drinking it once/day for a week. | C | MA59 b | |  |  |  |
| 15 | *Canthium lactescens* Hiern | Rubiaceae | KORBOO | **S**h | L | Hu | F | Dm | Itching (QANXOO/  CIITTO) | Chopping the young leaves & applying on the infected part. | R | MA132 | |  |  |  |
| 16 | *Capparis tomentosa* Lam. | Capparidaceae | GORRA GALLA | Li | R  Ba | Hu | F | O | Tooth ache  Wound (NAQARSA NAFAA) | Chewing & holding the root with infected tooth.  Chopping the root bark & applying on the infected part. | C | MA201 | | |  |  |
| 17 | *Carissa spinarum* L. | Apocynaceae | AGANSSAA | Li | L, R and  Root bark | Hu | F | O | Cold disease **(**GAMTOKKE).  Breast and  Stomach cancer (NAQARSSA)  Tooth ache  Evil eye (DRIYAA)  Skin cancer (NAQARSA NAFFAA) | Chewing 2 leaves once per day or Chewing the root bark with magado salt.  Chewing small part of the root and swallowing the juice.  Crushing the root and holding with the infected teeth or chewing the root with the infected tooth.  Chewing the root bark.  Crushing its root, combining it with the latex of *Euphorbia ampliphylIa* and applying on the wound. | C | MA113 | | |  |  |
| 18 | *Clematis hirsuta* Guill. & Perr. | Ranunculaceae | FIITTI | Li | L | Hu | F | O  Na & Dm | Swelling &  forming  wound on  the body  (LUXAA).  Asthma (GURRO OR SHIINQAA).  Head ache and coughing.  Gland TB (XANACHAA) | Crushing the leaves and applying on the affected part.  Chopping the leaves, making solution and applying a droplet through each nostrils**.**  Inhaling the crushed leaves to treat head ache and coughing.  Chopping the leaves, making s/n and giving 1 coffee cup 3 times a day for 1 week. | C | MA159 | | |  |  |
| 19 | *Clerodendrum myricoides* (Hochst.) Vatke | Lamiaceae | MARDHISSISA | Sh | L, R & St | Hu | F | Dm  &  O | Wound (MADAA)  Gonorrhea (DHUKUBA DHIRA)  Tooth ache | Chopping the leaves and applying on the wound.  Chopping the root, making s/n, boiling it with magado and giving ½ water glass 2 times a day for 3 days. Smelling the crushed leaves for 1 day also facilitate the treatment.  Brushing the teeth with its stick. | C | MA20 | | |  |  |
| 20 | *Clitoria ternatea* L. | Fabaceae | DINGATAGNA | Li | L &  R | Hu | F | O | Stomach cancer.  Snake bite or venom (IDDANSAA BOFAA).  Stomach and abdomen ache.  Sore in the mouth | Chopping the root and boiling it with water to give 2 coffee cup once a day for cancer patient.  Crushing the root with magado salt, making s/n and giving half to 1 coffee cup at once.  Chewing the root and the leaves.  Chewing the root for sore in the mouth. | R | MA191 | | | |  |
| 21 | *Clutia lanceolata* Forssk. subsp. *lanceolata* | Euphorbiaceae | KUTTAA DHIIGGA | Sh | R & L | Hu | F | O | Bloody Diarrhea (ALBAATTII).  Wound (MADAA)  Nerve disease (DHUKUBA ADUU) | Crushing the leaves and root together, making s/n and giving 1 coffee cup twice a day.  Crushing the root and placing on the wound.  Chopping the leaves, making s/n and giving 1 coffee cup at once. | C | MA127 | | | |  |
| 22 | *Combretum molle*  R.Br. ex G.Don | Combretaceae | RUKKENSAA | T | Rt  &  L | Hu  Hu | D  F | Na,Dm &  O  O | Evil eye  (DRIYAA)  Parasitic worms  Stomach ache (DHUKKUBA GARA) | Fumigating the  patient with dried and crushed root.  Crushing dried root with magado salt, making s/n, adding Camel milk and giving 1 water glass at once.  Chewing young leaves and swallowing the juice only. | C | MA52 | | | |  |
| 23 | *Commelina latifolia* Hochst ex A. Rich. | Commelinaceae | QAAYYO (WEFANQIR) | H | Latex | Hu | F | Dm | Wound | Dropping the latex on the infected part. | C | MA305 | | | |  |
| 24 | *Commicarpus plumbagineus* (Cav.) Standl | Nyctaginaceae | DHAKAAJII | Li | L | Hu | F | Dm | Wound | Chopping the leaves & applying on the wound | R | MA152 | | | |  |
| 25 | *Commiphora schimperi* (Berg) Engl. | Burseraceae | HAMEESSA DAALACHAA | Sh | L & R | Hu | F | O & Dm | Febrile illness (MICHII)  and tooth ache | Crushing the leaves and rubbing on the face.  Crushing the root with magado salt and holding with the infected tooth or heating the root and holding with the infected tooth. | R | MA127 | | | |  |
| 26 | *Crabbea velutina* S.Moore | Acanthaceae | CIRRECHA/ CIRRALLE | H | L & R | Hu | F | O | Stomach ache (GARAA DHUKKUBAA)  Itching (QANXOO) | Chewing 2 leaves with magado salt once when there is feeling of ache.  Chopping the leaves and applying on infected part. | R | MA94 | | | |  |
| 27 | *Crotalaria lachnophora* Hochst. ex A.Rich. | Fabaceae | QORSA  DIREYAA | Sh | Rt | Hu | F | Dm | Evil eye  (pain of all  parts of the  body)  - DRIYAA | Crushing the root,  boiling it & washing part of the body where pain is feeling without touching the ground with legs. | R | MA236 | | | |  |
| 28 | *Croton macrostachyus* Hochst. ex Delile | Euphorbiaceae | MOKONNIISA | T | Ba,  Ba  L  Latex  R | Hu  and | F | O | Tooth ache  Swelling  and forming  deep  opening (LUUXAA)  Cold disease (GAMTOKKEE)  Gonorrhea (DHUKUBA DHIRA)  Amoeboid (MAGENNAA) and Gonorrhea (DHUKKUBA  DHIIRAA)  Wound and tetanus  Kidney infection  Ring worm (BARRILLE)  Stomach ache  Gonorrhea (DHUKUBA DHIRA)  Hepatitis  Shivering and abnormal breathing (CUMA’A) | Holding/ chewing internal part of the bark with the infected tooth.  Crushing the internal bark, making thick s/n and adding to infected part.  Chopping root bark, making s/n, boiling, adding honey and giving 1 tea cup 2 times a day for 3 days.  Crushing internal part of the bark with its leaves, making s/n, boiling and giving 2 water glass 2 times a day for 3 days.  Chopping internal part of its bark, making s/n, adding honey and giving 1 - 2 coffee cup 2 times a day for 3 days.  Dropping the latex on the wound or infected part.  Crushing the leaves, making s/n and giving 2 coffee cups of the s/n for tetanus.  Crushing the leaves and internal part of the bark, making s/n and giving 3 coffee cups once.  Applying the latex on the infected skin once per day for 3 days.  Chopping the root, making s/n and giving 1 coffee cup once.  Crushing root bark, making s/n, boiling and giving 1 water glass 2 times a day for 3 days.  Chopping the leaves with the leaves of *Calpurnia aurea*, making s/n and giving one coffee cup for the patient at once.  Chopping inner bark, making s/n and giving 1 tea cup 2 times a day for 3 days. | C | MA137 | | | |  |
| 29 | *Cucumis pustulatus*  Naud. Ex Hook.f. | Cucurbitaceae | HAADHATU | Li | Rt | Hu | F | O | TB & sharp  pain on  sides of the  body  (DHUKKUBA  SOMBAA FI  WARRANA) | Chewing the root or  crushing the root,  making s/n &  drinking one coffee  cup daily until cured. |  | MA306 | | | |  |
| 30 | *Datura stramonium*  L. | Solanaceae | QOBBOO  ARDDAA | H | L | Hu | F | O | Rabies  (DHUKKUBA  SAREE) | Pounding the leaves, making s/n & giving 1-2 coffee cup for  adult humans and half of it for children | C | MA307 | | | |  |
| 31 | *Dioscorea schimperiana* Kunth | Dioscoreaceae | BAROODAA | Li | R | Hu | Dr | Dm | Dizziness & adding beauty in females (DADHABUMAA FI BAARREDUMAA NADHOOTTA) | Digging out the root, drying it, crushing and fumigate the patient. | C | MA31 | | | |  |
| 32 | *Dodonaea angustifolia* L.f. | Sapindaceae | DHITACHAA | Sh | L & St | Hu | F | O | Cold disease  (GAMTOKKEE)  Tooth ache | Crushing the leaves, making s/n and giving 1 water glass of it for the patient.  Brushing the teeth with its stick. | C | MA30 | | | |  |
| 33 | *Dovyalis abyssinica*  (A.Rich.) Warb | Flacourtiaceae | DHUGOO | Sh | L  Ba | Hu | F | O | Cold disease  (GAMTOKKEE)  Diarrhea in children | Pounding the leaves, making s/n & drinking one coffee cup.  Chewing the internal bark. | R | MA 73B | | | |  |
| 34 | *Ehretia cymosa* Thonn. | Boraginaceae | URAAGGA | T | L  Ba | Hu  Hu | F  F | Dm  O | Breast swelling (NAQARSSA MUCHA).  Cold disease  (GAMTOKKE).  Inflammation of children’s  Mouth (WAAN  AFAANII) and overflow of blood during menstruation.  Stomach ache in children (DHUKKUBII GARA IJOOLEEN)  Febrile illness (MICHII),  Severe headache (BOCAA)  Disturbed menstruation cycle. | Chopping the leaves with sugar & giving 2 coffee cups of its solution twice/ day.  Chopping young leaves, mixing it with honey and giving one tea cup for the patient for 3 days.  Chopping the leaves, making s/n and giving ¼ litre for the patient.  Chopping the leaves, making s/n and giving half to 1 coffee cup of it at once.  Crushing this part, making s/n, boiling, adding butter or milk and giving 1 tea cup once per day for 3 days. | C | MA308 | | | |  |
| 35 | *Ekebergia capensis* Sparrm | Meliaceae | ANONNU | T | Ba | Hu | F | O | Stomach cancer | Crushing internal bark of this plant with that of *Albizia schimperiana*  bark, making s/n and giving one water glass 2 times a day for one day. |  | MA143 | | | |  |
| 36 | *Endostemon tenuiflorus* (Benth.) M. Ashby | Lamiaceae | HAXAAWII | H | R | Hu | F | O | Gonorrhea (DHUKUBA DHIRA) & amoeboid (MAGEANA) | Crushing the root, boiling it with butter giving 1 coffee cup of it for the patient daily for 3 days. | C | MA60 | | |  |  |
| 37 | *Entada leptostachya* Harms | Fabaceae | HANDADDAA | Sh | R | Hu | F | O | Hepatitis (TIRUU) | Crushing the root with magado salt and giving 3 coffee cup of its s/n once per day. | R | MA97 | | |  |  |
| 38 | *Erythrina brucei**  Schweinf | Fabaceae | WALEENAA | T | Ba | Hu | F | O | Tooth ache  (DHUKKUBA  ILKAA) | Chewing internal part of stem bark if  possible with *magado* salt. | R | MA359 | | |  |  |
| 39 | *Eucalyptus*  *camaldulensis*  Dehnh. | Myrtaceae | BAARGAMO  DIIMA | T | L | Hu | F | O | Stomach  ache  (DHUKKUBA  GARRA) | Chewing the young  leaves & swallowing it. | R | MA309 | | |  |  |
| 40 | *Euclea divinorum* Hiern | Ebenaceae | MI’EESSA | Sh | R  Ba | Hu | F | O | Stomach ache (DHUKKUBA  GARRA)  Diarrhea (ALBAATTII)  Gonorrhea  (DHUKKUBA  DHIIRAA) | Crushing the root, making s/n, boiling and giving 1 coffee cup once/day.  Crushing inner bark, making s/n, boiling, adding milk or butter and giving 1 water glass 2 times a day for 3 days.  Crushing internal bark, making s/n, boiling, adding butter and giving one coffee cup 2 times a day for one day. | C | MA47 | | |  |  |
| 41 | *Euphorbia ampliphylIa* Pax | Euphorbiaceae | HADAAMAA | T | Latex | Hu | F | Dm  O | Wound (NAQARSSAA)  Increased  bile  production  (malaria)  (HADHOOTTU)  Tooth ache. | Taking the latex, combining it with the crushed *Carissa spinarum* root and applying on the wound.  Taking small amount of the latex, adding water and giving 1 water glass 2 times a day for the patient.  Applying the latex on the infected tooth.  Applying the latex on the infected part. | C | MA48 | | |  |  |
| 42 | *Euphorbia*  *depauperata* A.  Rich. | Euphorbiaceae | GURII | H | La | Hu | F | Dm | Skin rash &  ring worm  (KORMOMMAAN FI ROOBBII) | Taking the latex &  applying on the  infected part. |  | MA310 | | |  |  |
| 43 | *Fagaropsis angolensis* (Engl.) Dale | Rutaceae | SISSAA | T | Ba, R, L & St | Hu | F | O | Gonorrhea (DHUKUBA DHIRA) & regulating menstrual cycle.  Tooth ache  (DHUKUBA ILKKA)  Stomachache (GARA DHUKUBU)  Coughing (QUFAA/YIIKEE)  Shivering  and  abnormal  breathing  (CUMA’A) | Chopping the bark, boiling it with water, and giving 2 coffee cups for the patient twice a day.  Crushing these parts and giving 2 coffee cups solution for an adult human patient once a day or brushing the teeth with its stick.  Chopping the root, making s/n and giving one coffee cup at once.  Chopping internal part of the bark, making s/n and giving one coffee cup 2 times a day for one week.  Chopping its leaves with the leaves of *Viscum tuberculatum* *,* making s/n and giving one coffee cup 2 times a day for 3 days. | C | MA49 | | |  |  |
| 44 | *Flacourtia indica*  (Burm. f.) Merr. | Flacourtiaceae | HAGALAA | T | Ba | Hu | F | O | Swelling of  part of the  body due to  cold disease  (GAMTOKKE) | Chopping bark of the stem, making s/n drinking one coffee cup 2 times a day until the patient is cured. | C | MA119 | | |  |  |
| 45 | *Foeniculum vulgare*  Miller | Apiaceae | KALKALA YKN INSILAALA | H | Rt | Hu | F | O | Stomach ache  (DHUKKUBA  GARRA) | Pounding the roots,  making s/n & giving a coffee cup. | R | MA311 | | |  |  |
| 46 | *Fuerstia africana* T. C. E.Fr. | Lamiaceae | QAYYAA DURAA | H | L | Hu | F | Dm | Itching (QANXOO/CIITTO).  Wound  (MICHI) | Crushing the leaves and adding saliva to apply on the affected part.  Crushing the leaves and putting on the wound. Crushing the leaves with the leaves of *Ocimum* *spicatum* and giving half a coffee cup to the patient and polishing some amount on the face and drinking the remaining. | C | MA122 | | |  |  |
| 47 | *Gardenia ternifolia* Schumach.& Thonn. | Rubiaceae | GAAMBELLO | Sh | Ba | Hu | F | O | Severe headache (BOCAA), fever and disturbed menstrual cycle | Chopping internal part of the bark, making s/n, boiling, adding milk or butter and giving 1 water glass 2 times a day for 3 days. | C | MA219 | | |  |  |
| 48 | *Gerbera piloselloides* (L.) Casso | Asteraceae | ANQAREESSA | H | L | Hu | F | O | Stomachache (GARA DHUKUBU) | Chopping the leaves, making s/n and giving one coffee cup at once. | C | MA312 | | |  |  |
| 49 | *Gnidia stenophylla* Gilg. | Thymelaceae | ARSSAA | H | R | Hu | F | O | Gonorrhea (DHUKKUBA  DHIIRAA) | Crushing the root, making s/n, adding milk, staying in the sunlight for 3 hours and giving one coffee cup only | C | MA24 | | |  |  |
| 50 | *Gymnanthemum amygdalinum* (Delile) Sch.Bip. | Asteraceae | EEBIICHA | Sh | R  & L | Hu | F | O | Cold disease  (GAMTOKKEE)  Tooth ache  Urine retention (BOKKOKSAA FI DHIIBIINSA FIINCAANII) | Chopping the root, making s/n, adding honey and giving 2 coffee cups per day for a week.  Chewing the root and holding with the diseased teeth.  Chopping the leaves, making s/n and giving one coffee cup. | C | MA156 | | |  |  |
| 51 | *Haplocoelum*  *foliolosum* (Hiern)  Bullock | Sapindaceae | CANAA | Sh | Se | Hu | F | O | Ascaris  (MAAGAA) | Chewing a handful of ripened seeds and swallowing it. | R | MA216 | | |  |  |
| 52 | *Hordeum vulgare* L. | Poaceae | GARBUU | H | Se | Hu | Dr | O | Broken bones & worn out  tissues (LAFEE  CABAA FI NAFAA DADHABAA | Slightly toasting and grinding the seeds, preparing soup by adding milk or butter and drinking it as necessary. | R | MA314 | | |  |  |
| 53 | *Hypnum sp*. Hedw  (Moss) | Hypnaceae  (Bryophyte) | BIIQIILTUU JIIRMEE | Epiph. | The whole part | Hu | Roasted | Dm | Itch (CACAA OR CHIFEE) | Roasting these plants on a plate, powdering them, adding butter and applying on the infected part. |  | MA344 | | |  |  |
| 54 | *Isothecium* sp. Brid (Moss) | Lembophyllaceae  (Bryophyte) | ARII MUKKAA | Epiph. | The whole | Hu | Roasted | Dm | Itch (CACAA OR CHIFEE) | Roasting these plants on a plate, powdering them, adding butter and applying on the infected part. |  | MA345 | | |  |  |
| 55 | *Justicia schimperiana*  (Hochst. ex Nees) T. Anders. | Acanthaceae | CIIGGAA | Sh | L | Hu | F | O | Hepatitis  (BEKEKKO  YKN BIRTEE) | Chopping young leaves, making s/n & drinking half of water glass at once. | R | MA315 | | |  |  |
| 56 | *Kalanchoe*  *densiflora* Rolfe | Crassulaceae | HANCULLEE | H | L | Hu | F | Dm | Rheumatic  pain (NAFAA  BOCU) | Heating the leaves on fire & put on where pain is feeling. | C | MA279B | | |  |  |
| 57 | *Lagenaria siceraria* (Molina)Standl*.* | Cucurbitaceae | BUQQII | Li | Leaf latex | Hu | F | Er | Ear disease (DHUKKUBA GURRA) | Dropping the latex into the ear canal | C | MA354 | | |  |  |
| 58 | *Lippia adoensis** Hochst. ex Walp. var. *adoensis* | Verbenaceae | UDDOO | Sh | L | Hu | F | Dm | Body swelling | Chopping the leaves and mixing with water to wash the infected part of the body. | C | MA04 | | |  |  |
| 59 | *Millettia ferruginea**  (Hochst.) Back | Fabaceae | DHAADHATU | T | L  Se | Hu | F  D | O  Dm | Cold & flee  infection  (GAMTOKKE  FI MUJALEE) | Chopping the leaves, making s/n, adding honey and drinking one water glass at once for cold.  Crushing the seeds,  making thick s/n and applying on the  infected toes &  fingers for flee  infection. | C | MA173 | | |  |  |
| 60 | *Momordica foetida*  Schumach. | Cucurbitaceae | SURUPHAA  BOFAA | Ch | Rt | Hu | F | O | Rabies &Gonorrhea  (DHUKKUBA  SAREE FI DHUKKUBA  DHIIRAA) | Pounding the roots,  making s/n drinking  one coffee cup at once. | C | MA317 | | | |  |
| 61 | *Nuxia congesta* R.Br. ex Fresen | Loganiaceae | UDDESSA | T | L | Hu | F | O | Cold disease  (GAMTOKKE) | Pounding the leaves with the leaves of *Asparagus africanus*,  making s/n &drinking one water glass at once. | R | MA158 | | | |  |
| 62 | *Ocimum spicatum* Deflers | Lamiaceae | HANCABBII | Sh | L & R | Hu | F | Dm | Febrile illness (MICHII).  Wound (MADAA) | Chopping the leaves, making solution and rubbing on the face and hands for febrile illness.  The residue is used to treat the wound. | C | MA03 b | | | |  |
| 63 | *Ocimum urticifolium* Roth.S.Lat. | Lamiaceae | HANCABII | Sh | L | Hu | F | O & Dm | Febrile illness (MICHII)  Stomach ache | Chopping the leaves, squeezing the juice, rubbing on the face and other body parts.  By making solution with some of the crushed leaves half of a coffee cup is given to the patient.  Chewing the leaves and swallowing the juice. | C | MA03 a | | | |  |
| 64 | *Olea europaea* L. subsp. *cuspidata* (Wall. ex G.Don) Cif. L’Olivicoltore | Oleaceae | EJEERSSA | T | L  Ba  St | Hu | F | O | Bloody diarrhea and measles  Tooth ache | Chopping the leaves, making s/n and giving 1 coffee cup for the patient. It is possible to use its boiled form. Or  Crushing internal part of the bark, making s/n, boiling and giving one coffee cup for 3 days.  Brushing the teeth with its stick. | C | MA126 | | | |  |
| 65 | *Olinia rochetiana* A. Juss. | Oliniaceae | QADIDA DALACHA | T | L | Hu | F | O | Headache (MATAA BOWUU) | Chopping the leaves making s/n and giving half of a coffee cup for the patient at once. |  | MA318 | | | |  |
| 66 | *Osyris quadripartita*  Decn. | Santalaceae | WAATOO | Sh | L  Rt | Hu | F | O | TB  (DHUKKUBA  SOMBAA) | Pounding these parts, making s/n & drinking one water glass daily for a month. | R | MA224 | | | |  |
| 67 | *Pappea capensis* Eckl. & Zeyh. | Sapindaceae | BIIQQAA | T | Ba & L | Hu | F | O | Hepatitis (BIIRTEE)  Stomach pain after birth (MAARAA)  Tooth ache  Gonorrhea (DHUKKUBA  DHIIRAA) and disturbed menstrual cycle.  Breast cancer  Snake venom and severe headache (BOCAA) | Crushing inner part of the bark and adding a coffee cup of honey and delay for three days then giving one coffee cup for the patient.  Crushing the bark, making s/n, boiling it with butter and giving ¼ liter of it for the patient.  Chopping inner part of the bark and holding with the diseased tooth.  Crushing internal part of the bark, making s/n, boiling, adding milk or butter, and giving 1 water glass 2 times a day for 3 days.  Chopping inner part of the bark, making s/n, boiling, adding sugar and giving 1 water glass 2 times a day for 5 days.  Chopping the leaves and inner bark together, making s/n and giving 1 coffee cup 2 times a day for 3 days. | C | MA05 | | | |  |
| 68 | *Pavetta abyssinica* Fresen. | Rubiaceae | KOMOQORSSA | Sh | Rt | Hu | F | O  Dm | Tooth ache  & wounded  Cancer/skin cancer  (DHUKKUBA  ILLKANI FI  CACASSAA) | Chewing the root for tooth ache.  Pounding the root & put on the infected part. | R | MA18 | | | | |
| 69 | *Pavetta oliveriana* Hiern | Rubiaceae | KOMOQORSSA | Sh | L | Hu | F | O & Nas | Urine  retention  (DHIDIINSA  FINCAANII) | Chopping the leaves, making s/n & drinking one coffee cup at once or smelling the chopped leaves. | C | MA18 | | | | |
| 70 | *Pittosporum viridiflorum* Sims | Pittosporaceae | IRBAA YKN  BUDICHAA | Sh | R & St | Hu | F &  Dr | O & Dm | Evil eye  (DRIYAA)  Tooth ache | Fumigating the patient with the dried root and spitting the chewed root on the face.  Brushing the teeth with its stick. | R | MA319 | | | | |
| 71 | *Plectranthus tenuiflorus* (Vatke) Agnew | Lamiaceae | BARBARREESSA | Ch | R | Hu | F | Dm | Wound | Chopping the root and applying on the wound. | C | MA235B | | | | |
| 72 | *Podocarpus falcatus* (Thunb.) R.B. ex Mirb. | Podocarpaceae | BIRBIRSSA | T | Ba | Hu | F | O | Gonorrhea (DHUKKUBA  DHIIRAA) | Crushing the internal part of the bark with the bark of *Croton macrostachyus,* making s/n, boiling and giving 1 water glass 2 times a day for 3 days. | C | MA114 | | | |  |
| 73 | *Polyscias fulva*  (Hiern) Harms | Araliaceae | GUDDUBA | T | L | Hu | F | O | Amoebiasis  (MAGEANA) | Chopping the leaves,  making s/n and  drinking one coffee  cup. | R | MA320 | | | |  |
| 74 | *Polysphaeria parvifolia* Hiern | Rubiaceae | MIQQEE | Sh | L | Hu | F | Dm | Wound  (MADAA) | Pounding the leaves  and put on the wound | R | MA164 | | | |  |
| 75 | *Premna schimperi* Engl. | Lamiaceae | XULANGGEE | Sh | St  L | Hu | F & Dr | O | Tooth ache and testifying milk  Stomach ache | Brushing the teeth with its stick and fumigating milk container to give good taste.  Chewing young leaves and swallowing the juice. | C | MA29 | | | |  |
| 76 | *Rhoicissus revoilii* Planch | Vitaceae | LAALLUU | Li | R | Hu | F | Dm | Tooth ache | Chopping the roots and applying on the infected tooth | C | MA07 | | | |  |
| 77 | *Rhus natalensis*  Krauss. | Anacardiaceae | DABOOBESS  AA | Sh | L | Hu | F | O | Snake bite  (IDDANSAA  BOFAA) | Chewing a handful of its leaves | R | MA335 | | | |  |
| 78 | *Rhus vulgaris* Meikle | Anacardiaceae | DABOBEESSA | Sh | L | Hu | F | Dm | Itching (QANXOO/CIITTO)  Ring worm **(**BIIFAA YKN ROBBII) | Crushing the leaves with megado salt applying on the affected part.  Crushing the leaves, making s/n and giving 1 coffee cup 2 times a day for 3 days. | C | MA134 | | | |  |
| 79 | *Rhynchosia ferruginea* A.Rich. | Fabaceae | KALLAALTU | Ch | L | Hu | F | O | Stomach ache (BOKOKA) | Chopping the leaves with magado salt, making s/n and giving one coffee cup for the patient. | C | MA86 | | |  |  |
| 80 | *Ricinus communis* L*.* | Euphorbiaceae | QOOBBOO | H | L | Hu | F | O | Urine retention  Rabies  (DHUKKUBA  SAREE) | Pounding its leaves  with the leaves of  *Croton*  *macrostachyus*,  making s/n and giving one coffee cup. | C | MA322 | | |  |  |
| 81 | *Rumex abyssinicus*  Jacq. | Polygonaceae | DHANGAGO | H | Rt | Hu | F | O | Gonorrhea  (DHUKKUBA  DHIIRAA) | Pounding the roots,  boiling, adding butter and drinking one water glass daily until cured. | R | MA323 | | |  |  |
| 82 | *Schrebera alata*  (Hochst) Welw | Oleaceae | DHAMEE | T | St & Ba | Hu | F | O | Tooth ache  and throat  pain (NAQARSAA  ILKAA FI QALXAA) | Brushing the teeth with its stick or chewing internal part of stem bark and not swallowing the juice for tooth ache but swallowing for throat pain. | R | MA325 | | |  |  |
| 83 | *Solanum dennekense* Dammer | Solanaceae | HIIDII | Sh | R & Se  L & R  Fr | Hu | F | O | Goiter  Swelling  and forming  deep  opening (LUUXAA)  Tonsilites (SIILLISSA)  Stomach ache  Gland TB (XANACHAA) | Crushing roots and seeds, making s/n and giving ¼ of coffee cup of its solution to the patient.  Chopping the leaves and root together, making s/n, introducing through the opening and closing the opening with the residue.  Crushing the root, making solution and giving 1 water glass at once.  Chewing the root bark with magado salt.  Taking the juice of the fruit, making s/n and giving 1 water glass of it once a day for 1 week. | C | MA78 | | |  |  |
| 84 | *Solanum incanum* L. | Solanaceae | HIIDDI | Sh | Root Ba  R | Hu  Hu | F  F | O  O | Stomach ache (DHUKKUBII GARAA)  Hepatitis (TABBIISA YKN TIRUU) | Chopping the root bark, making s/n and giving 1 coffee cup for the patient at once.  Crushing the roots, making s/n and giving one coffee cup 2 times a day for a week. | C | MA327 | | |  |  |
| 85 | *Steganotaenia araliacea* Hochst. | Apiaceae | LUQAALUQQE | T | L  &  R | Hu | F | O | Over flow of menstruation | Chopping the root, making s/n, boiling and giving ¼ liter 2 times a day. | R | MA237 b | | |  |  |
| 86 | *Teclea borenensis* M.Gilbert | Rutaceae | HADHEESSA | Sh | R & L | Hu | F | O | Stomach ache (DHUKKUBIGARAA).  Diarrhea (GARAA KAASAA)  Breast cancer (NAQARSSA HAARMEE)  Evil eye | Chopping the root, making s/n, boiling it and giving half of coffee cup once per day.  Chewing the leaf with magado salt once per day.  Crushing inner part of the root, making s/n and giving one coffee cup 3 times a day for 1 week.  Chopping the root, making s/n, boiling and giving 1 tea cup per day for 3 days. | C | MA275 | | |  |  |
| 87 | *Teclea salicifolia* Engl. | Rutaceae | HADHEESSA | Sh | L & St | Hu | F | O | Diarrhea (ALBAATTII).  Wound (CACCA)  Hepatitis (BIIRTEE)  Stomach ache  Tooth ache (DHUKUBII IILKAAN) | Crushing the leaves giving 1 coffee cup of its solution for the patient.  Heating the leaves on fire, adding butter and putting on the wound.  Chopping the leaves, making s/n and giving one coffee cup at once.  Brushing the teeth with its stick or  Chopping the leaves and holding it with the infected tooth. | R | MA77 |  |  |  |  |
| 88 | *Terminalia brownii* Fresen | Combretaceae | BIDHEESSAA | T | Ba | Hu | F | O | Hepatitis/ BIIRTEE  To narrow the uterus after birth.  For beautification | Chopping the bark with megado salt and 1 coffee cup solution is given for adult human patient/day.  Fumigation with dried stem for beautification. | C | MA70 |  |  |  |  |
| 89 | *Vachellia abyssinica* (Hochst. ex Benth.) Kyal. & Boatwr. | Fabaceae | HONDODDEE | T | R | Hu | D | Na & Dm | Evil eye  (DRIYAA) | Taking dried root parts & fumigating  the patient. | C | MA108 |  |  |  |  |
| 90 | *Terminalia prunioides* Law | Combretaceae | QOROBBOO | T | L | Hu | F | Dm | Itching (QANXOO/CIITTO) | Crushing the leaves and applying on the affected part. | R | MA64 |  |  |  |  |
| 91 | *Vangueria apiculata* K. Schum | Rubiaceae | BURURII | Sh | Root bumps  L | Hu | F | O  Na | Liver disease  Urine  retention  (DHIDIINSA  FINCAANII) | Chopping the root bumps, making s/n, and giving 1 tea cup.  Chopping the leaves, making s/n & drinking one coffee cup at once or smelling the chopped leaves. | R | MA87 |  |  |  |  |
| 92 | *Vernonia*  *auriculifera* Hiern | Asteraceae | REEJII | Sh | L  St | Hu  Hu | F  F | Dm  O | Wound & stop bleeding during injury (clotting)  (MADAA FI DHIIGAA DHABUF)  Amoebiasis  (MAGEANA) | Crushing the leaves and putting on the affected part.  Peeling the young  stem near the  meristem and  chewing it | C | MA329 |  |  |  |  |
| 93 | *Viscum tuberculatum* A. Rich. | Viscaceae | DHERTOO | Epi | St & L | Hu | F | O | Poisons, snake venom  Shivering  and  abnormal  breathing  (CUMA’A) | Chopping the whole parts, making s/n and giving 1 coffee cup 2 times a day.  Or Chopping its leaves with the leaves of *Fagaropsis angolensis* *,* making s/n and giving one coffee cup 2 times a day for 3 days. | C | MA330 |  |  |  |  |
| 94 | *Warburgia ugandensis* Sprague | Canellaceae | BIITTII | T | Ba | Hu | F | O | Internal cancer, stomach ache, weight loss and prolonged fever | Crushing internal part of the bark, making s/n, boiling and giving 1 tea cup 3 times a day for a week. | R | MA331 |  |  |  |  |
| 95 | *Withania somnifera* (L.) Dunal | Solanaceae | LALLAAAFFA | Sh | R | Hu | F & Dr | O | Snake venom (HADHAA BUTTE YKN BOFAA).  Stomach ache.  Evil eye | Chopping the root, making s/n & giving 1 - 2 coffee cup of it to the victim.  Chewing the root with magado salt.  Fumigating with the dried root. | C | MA248 |  |  |  |  |
| 96 | *Ximenia caffra* Sond. | Olacaceae | HUDHAA | Sh | Ba | Hu | F | Dm | Wound | Chopping the bark & applying on the wound. | R | MA186 |  |  |  |  |
| 97 | *Zanthoxylum chalybeum* Engl. | Rutaceae | GADDAA | Sh | Ba, R, Se, L & St | Hu  Ls | F  Dr | O | Tooth ache (NAQARSA ILKAAN).  Amoebiasis (MAGEANA) &  Typhus (GOGOSSA)  Hypertension  (DANFFA DHIIGAA)  Vertebral column pain (DHUKUBII DUBA) | Crushing the bark with magado salt and applying on the infected tooth or chewing the root or  brushing the teeth with its stick.  Crushing the seed, bark, and root, making s/n and giving 2 coffee cups/day for amoebiasis and typhus.  Chopping the internal part of the bark and the leaves together, boiling and drinking like tea or powdering the dried form of these parts and boiling as tea and drinking 1 - 2 tea cup 2 times a day.  Chopping inner part of the bark, making s/n, boiling it, adding butter and giving 1 tea cup 2 times a day for 1 week. | R | MA16 |  |  |  |  |
| 98 | *Ziziphus abyssinica* Hochst ex A. Rich. | Rhamnaceae | HUQUNQURA | T | Ba  &  L | Hu | F | O  Dm | Severe headache (BOCAA), fever and disturbed menstrual cycle  Wound (MADAA) | Chopping the bark, making s/n, boiling, adding milk or butter and giving 1 water glass 2 times a day for 3 days.  Chopping the leaves and applying on the wound. | C | MA217b |  |  |  |  |
